# Local Induction Melting Combined with Ultrasonic Melt Treatment: A Pilot Study on Porosity, Mechanical Properties, and Energy Efficiency in AlSi12 Castings

**DOI:** 10.3390/ma19204315

**Published:** 2026-10-13

**Authors:** Serouj Djabraian, Johannes Dettelbacher, Alexander Buchele, Fabian Teichmann, Sebastian Müller

**Affiliations:** 1 Chair of Casting Technology, Friedrich-Alexander-Universität Erlangen-Nürnberg, Dr.-Mack-Str. 81, 90762 Fürth, Germanyseb.mueller@fau.de (S.M.); 2 Faculty of Engineering Sciences, Ansbach University of Applied Sciences, Residenzstraße 8, 91522 Ansbach, Germany; johannesdettelbacher@yahoo.de

**Keywords:** foundry electrification, induction melting, ultrasonic melt treatment, AlSi12, porosity, X-ray computed tomography, mechanical properties, energy efficiency

## Abstract

Light metal foundries consume large amounts of energy and rely mainly on gas-fired melting, which is inefficient and promotes porosity caused by dissolved hydrogen. This pilot study examines local induction melting combined with ultrasonic melt treatment (UST) as a decentralized and electrified alternative. AlSi12 was cast with varied mold temperature, casting temperature, UST duration and remelted fraction. Porosity was characterized by optical microscopy in 2D and, for a subset of specimens, by X-ray computed tomography in 3D, and the mechanical behavior by tensile testing. An experimentally benchmarked simulation was used to estimate the energy demand for larger batch sizes. Including the energy required for cooling, the measured laboratory efficiency was 7.9% to 14.9%, and the simulation predicts 41.3% for 100 kg, or 48% with a preheated crucible, against 53.1% derived for industrial induction furnaces. Specimens treated with UST showed a lower mean porosity, 0.56% against 2.14% in optical microscopy and 0.153% against 0.367% in computed tomography, where the pore count was 73% lower at almost unchanged pore size. Their mean strain at fracture was 45.5% higher and their mean toughness 53.2% higher, whereas the 4.4% higher mean tensile strength lies within the scatter of the measurements.

## 1. Introduction

**Figure 1:**
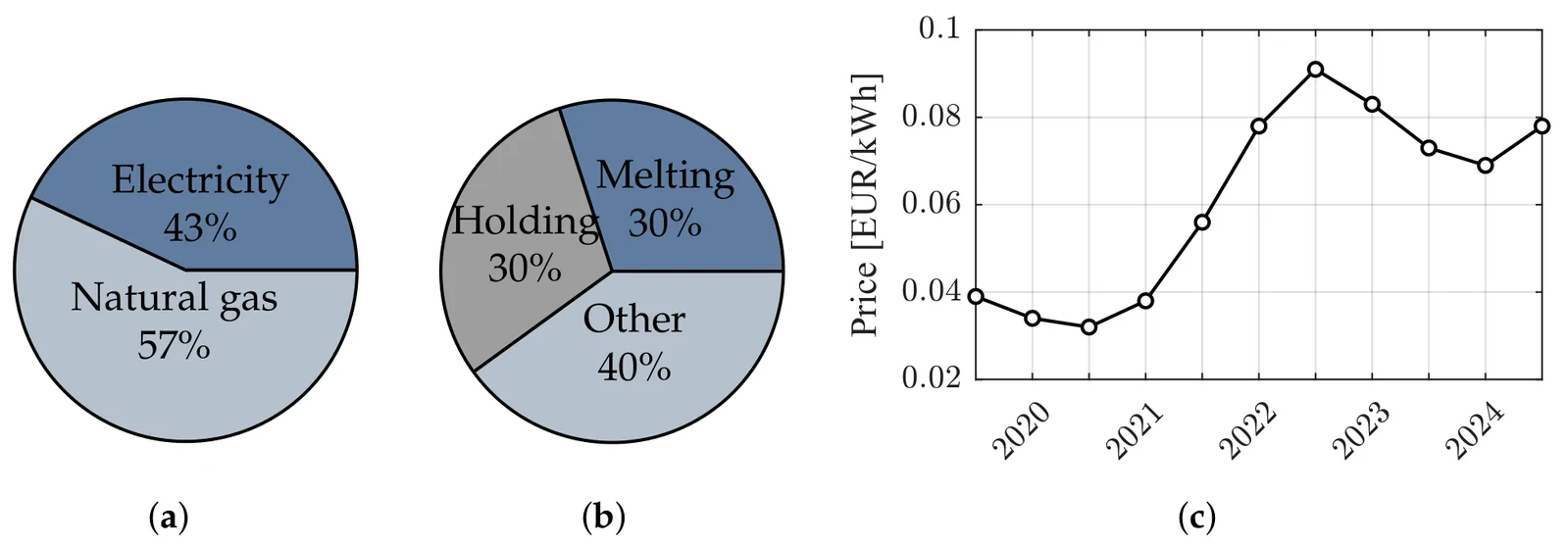
Energy use in German foundries and industrial natural gas prices. (**a**) Share of energy consumption of non-ferrous metal foundries [2,3]. (**b**) Energy requirements in a typical foundry [5]. (**c**) Industrial natural gas price in Germany [4].

The foundry industry supplies essential components for a wide range of industrial applications. Germany hosts one of the largest foundry sectors worldwide, and this sector is both economically important and a large consumer of energy. In 2019, German foundries used about 12.4 TWh of energy, corresponding to an estimated 5 million tonnes of CO_2_ equivalents [1]. Light metal and non-ferrous foundries accounted for around 4.7 TWh of this figure. Data from 2015, shown in Figure 1a, give an energy demand of 4.2 TWh for these foundries, of which fuel, mainly natural gas, covered roughly 57% [2], a share that remained at about 57% in 2022 [3]. Between 2020 and 2024, natural gas prices for industrial users in Germany fluctuated strongly, rising from a low of 0.032 EUR/kWh in 2020 to a peak of 0.091 EUR/kWh in 2022 and remaining well above the earlier level thereafter [4], as shown in Figure 1c. This dependence on fossil fuels raises operating costs and increases the environmental impact of foundry processes. Beyond carbon emissions and rising energy costs, the gas-fired melting systems commonly used in light metal foundries, such as reverberatory and crucible furnaces, suffer from poor thermal efficiency and typically operate at efficiencies of only 7% to 25% [5]. These systems also introduce moisture and other contaminants into the combustion environment, which increases the absorption of hydrogen in the melt. The result is higher porosity in the final casting, which often requires additional degassing steps and further energy [5]. Melting and holding together account for about 60% of the total energy use of a foundry, as shown in Figure 1b, which makes them the most energy-intensive stages of the process chain [5].

Induction melting offers a more efficient and cleaner alternative. Heat is generated electromagnetically rather than by combustion, either directly in the charge or in an electrically conductive susceptor surrounding it, so that no combustion gases are produced at the furnace and the power input can be adjusted directly [5]. Operating data from industrial aluminum melting furnaces give a mean specific energy consumption of 612 kWh/t for induction furnaces and 1664 kWh/t for reverberatory furnaces with cold air burners [6]. Related to the theoretical requirement of 325 kWh/t for heating aluminum to 760 °C given in [6], this corresponds to efficiencies of 53.1% and 19.5%. Induction furnaces also support melting under inert gases, which limits oxidation and the absorption of hydrogen during the processing of aluminum. The strong electromagnetic stirring inherent to induction technology homogenizes the melt. At the same time, it keeps the oxide skins of the charge in suspension, so that they cannot separate by flotation or sedimentation [7]. Studies on Al–Sn bearing alloys have shown that increasing the stirring intensity further improves the microstructure and produces finer and more spherical grains, less dendrite formation, and better corrosion resistance from denser passive films [8]. Modern induction systems also maintain a consistently high power factor through advanced frequency converters, and the stirring they provide makes it easier to use recycled material in the melt [9]. While induction melting is already widespread in iron and steel foundries, its use in aluminum foundries has so far been limited, mainly because natural gas has historically been cheaper than electricity. For melting aluminum specifically, induction furnaces are already more efficient in industrial use than furnace types based on burners [6]. In aluminum casting, for example, the Swedish foundry AB Lundbergs Pressgjuteri installed two medium frequency induction furnaces from Otto Junker GmbH for melting aluminum [9]. Induction melting systems are available across a wide range of capacities, from under 1 kg to several tonnes, and offer high flexibility across alloy systems including iron, steel, copper, aluminum, and precious metals. Induction melting can also benefit casting quality. Unlike gas-fired rotary and shaft furnaces, which introduce moisture during combustion and fresh air intake, induction furnaces remove this source of hydrogen uptake, although moist refractories, for instance after relining, can still release water vapor to the melt [7]. This reduces the need for downstream degassing operations, which typically rely on rotary impellers or chemical tablets and are difficult to automate and to integrate into digital process control. Induction melting therefore lends itself to decentralized and electrified foundries. Because induction melting involves closely coupled electromagnetic, thermal, and flow behavior, understanding and optimizing the process in detail typically requires numerical simulation. Many studies have simulated induction processes in molten metals using a range of methods. One approach focuses on melt flow driven by the Lorentz force [10,11], another on surface dynamics [12,13]. Simulations of induction heating and melting also frequently address the temperature distribution. One study built a coupled model of a nickel-based superalloy melt in an induction melting furnace [14], combining magnetic, hydrodynamic, and thermal behavior in COMSOL Multiphysics and comparing the numerical results with experimental data. A related study modeled the melting of steel in an induction furnace using MATLAB/Simulink [15], treating the thermal and the electrical behavior as separate subsystems and validating the results against theoretical predictions. Further work simulated the induction heating of carbon steel workpieces in COMSOL Multiphysics [16], calculating coil, magnetic field, and thermal parameters and checking them against experimental measurements. Other studies address the importance of temperature-dependent material properties in induction heating [17,18]. Most existing approaches use finite element method (FEM) techniques and software such as COMSOL Multiphysics. Simulation supports the optimization of the process, but the melt quality achieved in practice still depends on factors such as the dissolved gas content, which simulation alone cannot address. Even in processes based on induction, the melt often still contains dissolved hydrogen, which must be removed to ensure casting quality. Hydrogen is the only gas that dissolves readily in liquid aluminum but not in solid aluminum, which leads to gas porosity during solidification, a common defect that reduces the mechanical properties of the cast product [19]. The main methods used today for removing hydrogen are rotary degassing and chemical degassing tablets applied just before casting. Both rely on added substances and offer limited scope for automation or digital control. Rotary degassing units are common in the processing of aluminum alloys but have further drawbacks. Nominally inert gases often contain enough oxygen to oxidize the liquid metal, and impurities can enter through moisture absorbed by the rotor and shaft, or through unclean rubber or plastic gas tubing, so that the gas reaching the melt is often no longer inert [7]. A study on a secondary Al-Si alloy (AlSi9Cu3(Fe)) found that omitting the rotary degassing stage before casting had no measurable effect on tensile properties: yield strength stayed at around 150 MPa, with similar elongation to failure. Omitting the stage also gave a 75% reduction in the energy needed to reheat the melt, saving around 124 MWh annually and cutting CO_2_ emissions by about 1.5 t per furnace, or 32 t to 93 t per year [20]. These results question the assumed benefits of rotary degassing for mechanical performance and point to its environmental and economic costs. Ultrasonic melt treatment (UST) removes hydrogen without gas injection or chemical additives. Acoustic waves at high frequency create cavitation in the melt, which produces small bubbles that capture dissolved hydrogen and non-metallic inclusions and carry them to the surface. No oxygen or moisture is added, so the risk of secondary contamination is avoided. UST also improves melt homogeneity and refines the grain structure, which raises tensile strength and ductility. A study on A356 aluminum alloy using a titanium waveguide at 20 kHz showed that UST reduced the hydrogen content from 0.45 ppm to 0.14 ppm in a 0.2 kg melt within one minute, reaching a steady state density plateau and lowering porosity [21]. Research on AlSi9Cu3 alloy showed that UST produced fine globular α-Al grains, raising the ultimate tensile strength to 332 MPa and the strain to 2.9%, improvements of 50% and 480% over untreated castings [22]. Another study found that UST increases the alloy density to 98.5% of the theoretical value while degassing light alloys [23]. Applied with an immersed sonotrode directly before tilt casting, UST reduced the total pore volume measured by CT by 53% and increased the elongation by 17% [24].

Reducing the dependence on centralized gas-fired melting and on long holding times can improve both energy efficiency and casting quality. Continued use of fossil fuels also limits the integration of renewable energy sources and works against efforts to lower the carbon footprint of the sector. Induction melting and UST each address part of this problem. Induction melting improves energy efficiency and reduces hydrogen uptake from the furnace atmosphere, while UST removes hydrogen already dissolved in the melt. The two have so far been combined mainly in a contactless form, in which an induction coil above a melt held in an induction furnace excites cavitation without an immersed sonotrode, with a focus on grain refinement [25,26]. Immersed sonotrode UST has been applied to small melt volumes directly before casting, but with the melt prepared in a resistance furnace [24]. Whether immersed sonotrode UST can be integrated into local induction melting, and how the energy demand of such a unit relates to the resulting casting quality, therefore remains open. This work addresses this question with a pilot casting study on AlSi12 alloy that combines local induction melting with UST, varying mold temperature, casting temperature, UST duration, and the share of remelted material. Porosity is evaluated using optical microscopy in 2D and, for a subset of specimens, X-ray computed tomography in 3D, and the energy demand of the melting step is estimated with a simulation benchmarked against the laboratory unit and scaled to batch sizes of up to 100 kg.

## 2. Materials and Methods

### 2.1. Experimental Setup and Procedure

**Figure 2:**
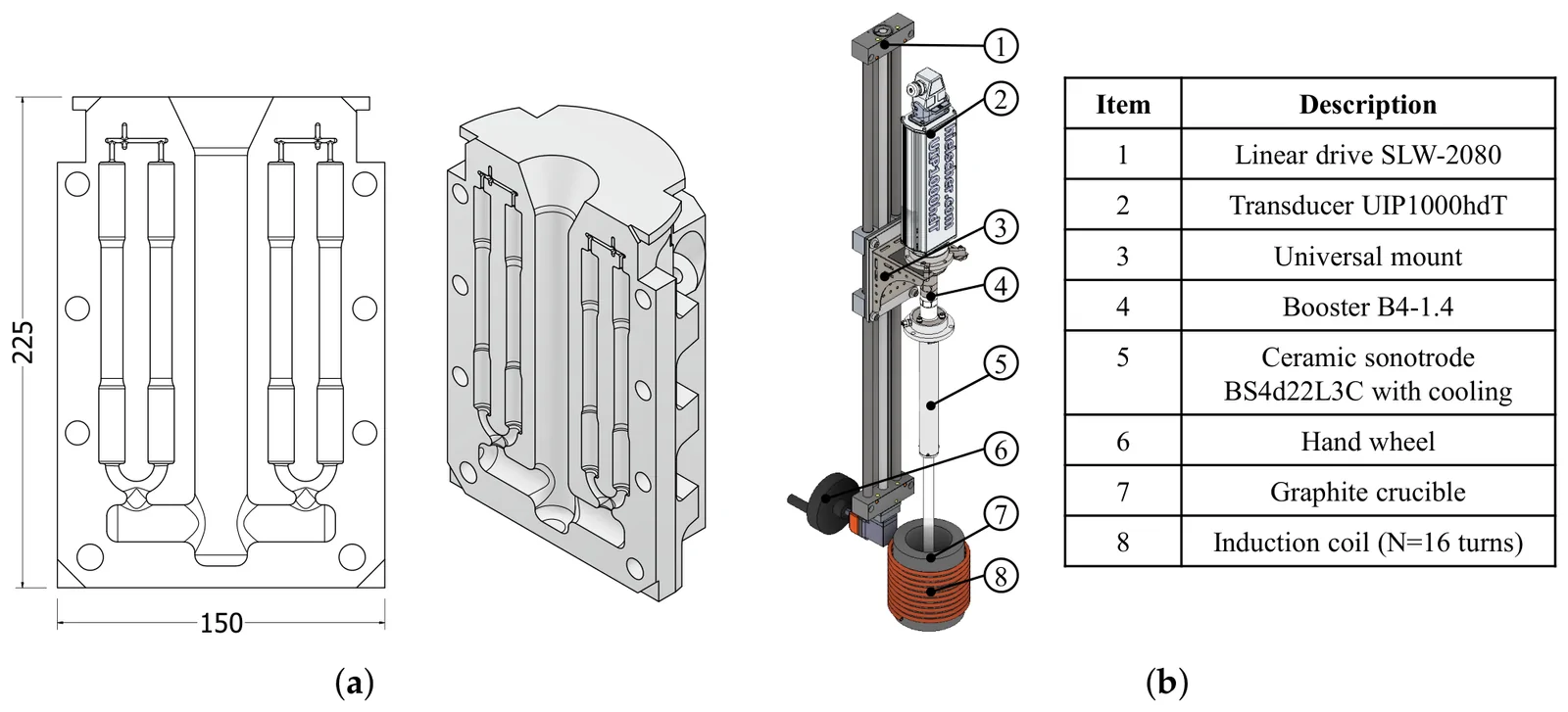
Experimental mold and setup schematics. (**a**) Steel mold half-model (Dimensions in mm). (**b**) Melting and ultrasonic treatment setup.

**Figure 3:**
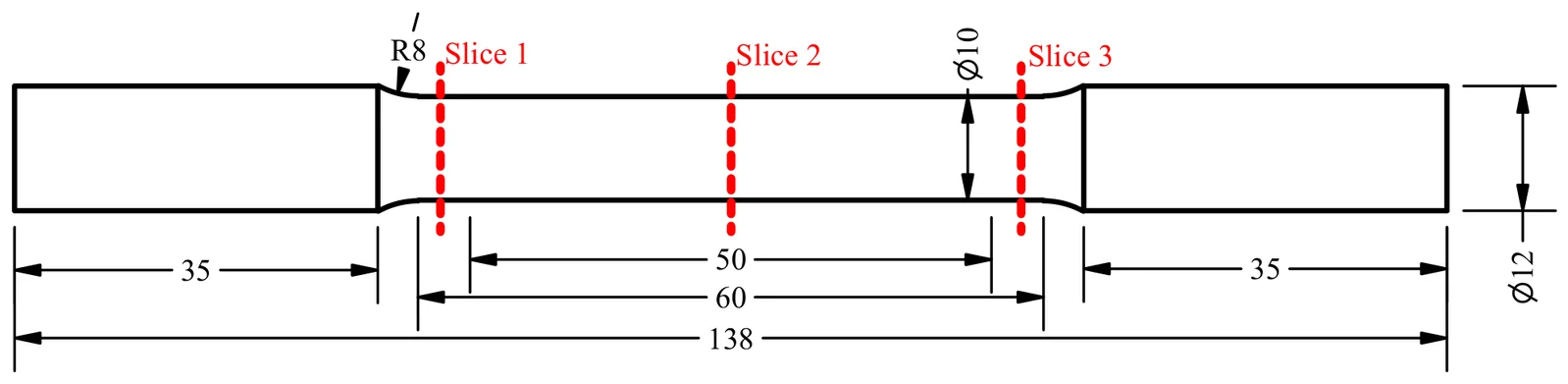
Round tensile test specimen conforming to DIN 50125-A10×50 (Dimensions in mm).

A series of casting trials was performed on AlSi12 aluminum alloy, combining local induction melting with UST under varied process conditions. Melting was carried out in a graphite crucible with a capacity of 1200 cm^3^ using an induction melting unit (MU1200, BluePower Casting Systems GmbH, Walzbachtal, Germany; 15 kW maximum power, operating frequency approximately 17.5 kHz), which gives fast heating with electromagnetic mixing. Each batch used 500 g of alloy, enough to produce four round tensile specimens per casting, poured by hand into a preheated tool steel mold (1.2343/H11, dimensions as per the schematic in Figure 2a). The pouring time was approximately 3 to 4 s per casting, which corresponds to a mean pouring rate of at most about 125 to 170 g/s for the batch of 500 g. The tensile specimens conform to DIN 50125-A10×50 [27], with dimensions detailed in Figure 3. Melt temperature was monitored continuously with a type S thermocouple at the crucible base. The induction coil was cooled with water through an external chiller (TAEevo Tech 020, MTA Deutschland GmbH, Nettetal, Germany). For trials involving UST, a UIP1000hdT ultrasonic processor (Hielscher Ultrasonics GmbH, Teltow, Germany) was used, fitted with a Si_3_N_4_ ceramic sonotrode tip suitable for light metal melts, operating at 20 kHz with a maximum power of 1000 W and set to 100% amplitude for all trials, corresponding to a maximum tip amplitude of 25 μm according to the manufacturer’s specification. As shown in Figure 2b, the ultrasonic unit was lowered into the melt to a fixed immersion depth of 20 mm with an SLW-2080 linear drive (igus GmbH, Cologne, Germany), controlled by hand via a handwheel and a mechanical position indicator on a rigid frame made of aluminum profiles. Pouring followed the end of UST by approximately 2 to 3 min in all trials with UST.

Four process parameters were varied: mold temperature $T_{\mathrm{mold}}$, casting temperature $T_{\mathrm{cast}}$, UST duration $t_{\mathrm{UST}}$, and the share of remelted alloy *R*, as detailed in Table 1. Initial trials, labeled T-01 to T-09, used virgin AlSi12 alloy without UST to isolate the effects of $T_{\mathrm{mold}}$ and $T_{\mathrm{cast}}$. Trials S-01 to S-03 then applied UST for 20 s, 40 s, and 60 s, respectively, while holding $T_{\mathrm{mold}}$ at 200 °C and $T_{\mathrm{cast}}$ at 700 °C with virgin alloy, to assess the effect of UST on porosity. Remelting was studied in trials R-01 and R-02, using 50% remelted AlSi12 alloy blended with virgin alloy at $T_{\mathrm{mold}}=$ 200 °C and $T_{\mathrm{cast}}=$ 700 °C, without and with 60 s of UST, respectively, to check for interactions between remelting and UST. Trials CT-01 to CT-04 used 100% remelted alloy under the same baseline conditions of $T_{\mathrm{mold}}=$ 200 °C and $T_{\mathrm{cast}}=$ 700 °C, with UST durations of 0 s or 20 s and two replicates each, for volumetric porosity characterization by computed tomography (CT). Each parameter combination in Groups T, S and R was cast once.

A schematic of the cast round tensile specimen is shown in Figure 3. The specimens conform to DIN 50125-A10×50, with a gauge diameter of 10 mm and an initial gauge length of 50 mm, chosen for uniform stress distribution and compatibility with cast aluminum alloys prone to internal defects. Each casting trial produced four specimens: three were tensile tested to fracture on a servo-electric universal testing machine (Walter + Bai AG; maximum load capacity 400 kN) using a video extensometer over the 50 mm gauge length, and the fourth was used for porosity characterization in 2D, sectioned transversely at three axial positions (Slice 1, Slice 2, and Slice 3), as shown in Figure 3. The sliced surfaces were ground and polished, with a final polishing step using 1 μm diamond suspension. The three sections capture the spatial variation in porosity within one specimen. They do not allow the variation between castings to be estimated, so the porosity analysis is descriptive.

Optical images of the polished cross sections were acquired with an optical microscope (LEXT OLS5500, Olympus Corporation, Tokyo, Japan) at 5× magnification, capturing the pore morphology across the full specimen cross section. Image analysis was carried out in ImageJ (version 1.54g) following a standardized workflow: images were first scaled to actual size using the known 10 mm specimen diameter as a reference, and then converted to 8-bit grayscale. Thresholding was applied manually on a per image basis to segment pores, since the gray value distribution differed between images due to variations in illumination and in the polished surface of each specimen, so that no single global threshold was applicable. The “Analyze Particles” function was then used with per image minimum area and minimum circularity filters, set by visual comparison of the resulting mask against the source image, to exclude noise, contaminants and polishing scratches while retaining pores. Overlay masks were generated on the segmented images, assigning unique colors and identifiers to each detected pore for visual verification. The segmentation therefore depends on operator judgment (Section 3.5). Pore metrics, including individual areas, circularities, and total porosity, defined as the ratio of cumulative pore area to specimen cross sectional area, were exported as CSV files for further analysis in MATLAB (R2023b), where global porosity averages and distributions were computed across the three sectional positions per specimen. To complement the optical analysis in 2D, the tensile specimens of castings CT-01 to CT-04 were characterized volumetrically by X-ray computed tomography. Two specimens were scanned per casting, for eight scans in total. The reconstructed volumes were exported as 16-bit HDF datasets with isotropic voxel sizes of approximately 0.0355 mm, depending on the individual scan. Volumetric image processing was carried out in MATLAB. Pores were segmented by intensity thresholding, with the threshold calibrated individually for each scan, in the range 46,000 to 48,000 of the 16-bit intensity range, by repeated visual comparison of the segmented volume against the reconstructed gray value data, which accounts for differences in intensity values between scans. Connected voids were labeled in three dimensions using 26-connectivity via the bwlabeln function of MATLAB, and the volume of each labeled feature was extracted using the regionprops3 function. From the volume $V_{p}$, the equivalent spherical diameter $d_{p}$, the diameter of a sphere with the same volume as the pore, was computed as

(1) $$d_{p}={\left(\frac{6V_{p}}{\pi }\right)}^{1/3}$$

Pore shape was not evaluated in 3D, because the surface area estimate required for a shape factor such as the Wadell sphericity becomes unreliable for the small pores that make up most of the population at the given voxel size. A minimum size filter was applied: features smaller than 20 voxels, corresponding to an equivalent spherical diameter of approximately 0.12 mm or about three voxel edge lengths (Equation (1)), were rejected as unresolved noise. Volumetric porosity ϕ was evaluated as the ratio of the cumulative pore volume to a common reference volume, a cylinder of 10 mm diameter and 60 mm length applied identically to all specimens. Because the optical analysis applies no comparable lower size bound, the two methods sample different parts of the pore population (Section 3.3).

### 2.2. Numerical Setup

**Figure 4:**
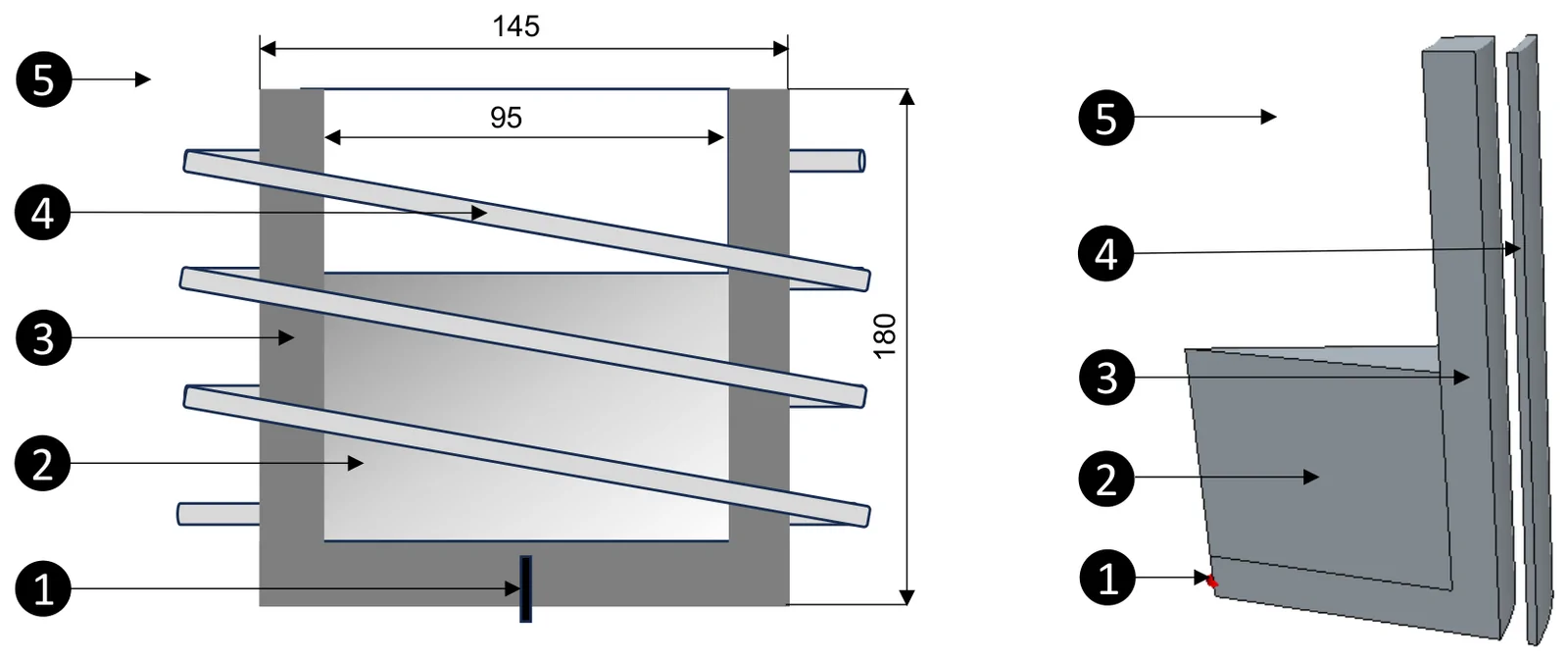
Simulation setup. (1) Temperature sensor, (2) aluminum, (3) graphite crucible (susceptor), (4) induction coil, and (5) air.

A simulation-based approach was used to estimate the process energy requirements. A model was set up in Simcenter STAR-CCM+ (version 17.04.007) to capture the electromagnetic and thermal phenomena of induction heating and melting, including the phase change in the alloy. First, a model of the existing melting unit, as described in Section 2.1, was created and benchmarked against experimental data. The model was then scaled up to represent larger industrial installations, which allows the performance under increased loads and volumes to be analyzed. The setup includes the following key elements: an induction coil, a crucible containing a solid aluminum charge, and a surrounding air domain. The geometry is based on data from the experimental setup as illustrated in Figure 4. To reduce complexity, the induction coil was modeled as a simplified hollow cylinder, and the aluminum was represented as a cylindrical volume contained within the crucible. The mass of the aluminum was calculated from the adjusted height of the aluminum column within the crucible. The simplified model does not account for the furnace geometry outside the coil and crucible, and losses were determined empirically from measurements. To reduce computational cost, geometric symmetry was used by simulating only a 15° sector of the full crucible geometry for the industrial scale model, as shown in Figure 4. This reduces mesh size and solver time substantially. The simulation also replicates the temperature sensor installed in the laboratory crucible, which was modeled as a single point probe that captures the local temperature at a defined cell centroid on the bottom of the crucible. The position of the virtual probe matches that of the actual sensor in the experimental setup, which enables direct comparison.

Several physics models from Simcenter STAR-CCM+ were used to simulate induction heating and melting. The Harmonic Balance FV Magnetic Vector Potential model captured electromagnetic effects by solving time-harmonic fields in the materials. Combined with the Excitation Coil model, this yields the current density in the coil, the resulting magnetic field, and the induced currents in the materials. Ohmic heating from eddy currents in the conductive crucible was also included, to calculate the heat generation from the Joule effect. For the aluminum alloy, a multiphase model accounted for the latent heat of fusion. Temperature was determined using segregated solid (crucible), fluid (air), and multiphase (aluminum) temperature models. Heat conduction, convection, and radiation were all considered for heat transport in the system. Radiation was modeled using a surface-to-surface approach with constant emissivity values for the crucible and melt surfaces. To simulate heat loss from the system, a heat loss coefficient was applied at the system boundaries, approximated from a series of measurements. The electrical losses of the coil were estimated from the same measurement series. The power measured at the melting unit was recorded every second and fed into the simulation as a CSV file. Since STAR-CCM+ requires a coil current rather than a power value, the power was first converted to the corresponding current, which was then read from the CSV file at each time step using a field function within the simulation. For scaling to larger plant sizes, a PI controller adjusted the current to reach the specified power output. The controller was implemented in STAR-CCM+ via reports and tuned by manual gain adjustment for stable convergence with minimal overshoot. The frequency was taken from the measured values and treated as constant, see Section 2.1. The material parameters of the individual components were stored as functions of temperature, using literature data and Thermo-Calc (version 2024b) (Al Databank 4.0). Table 2 lists the temperature-dependent thermophysical properties used. For the aluminum alloy, the thermal conductivity changes markedly at the melting point, with the liquidus and solidus temperatures both set to 577 °C. The simulation also relies on the following simplifications and assumptions:

- The density of the materials was assumed to be constant: 2700 kg/m^3^ for the aluminum alloy, 1900 kg/m^3^ for the crucible, and 1.184 kg/m^3^ for the air.
- The latent heat of fusion for the aluminum alloy was set to 560 kJ/kg.
- The emissivity was set to 0.8 for the crucible and 0.2 for the aluminum alloy.
- The initial temperature for all materials was set to 25 °C.
- The air properties used the default STAR-CCM+ values (heat capacity = 1003.62 J/(kg K); thermal conductivity = 0.0260305 W/(m K)).

Because the density of the air was held constant, buoyancy driven flow does not develop in the air domain. Free convection at the outer surfaces is therefore not resolved by the model and is instead covered by the empirical heat loss coefficient applied at the system boundaries. Meshing was performed automatically, using a trimmed cell mesher with prism layers. The simulation used a time step of one second, with the default solver settings retained. The quality of this numerical approach is assessed against the experimental measurements in the following section.

## 3. Results and Discussion

### 3.1. Energy Requirements

During melting, aluminum is heated until it reaches its melting point, changes phase to become a liquid, and is then superheated. The theoretical energy demand, excluding furnace efficiency, comprises these three stages: heating the solid to its melting temperature, melting at constant temperature, which requires the latent heat of fusion, and superheating the liquid to a target temperature. The total theoretical enthalpy change follows from the latent heat of fusion, the specific heat capacity, the temperature range, and the mass. Using the material data given in Section 2.2, the theoretical energy requirement to heat the alloy to 720 °C is 356 kWh/t, from Equation (2). All efficiencies reported in this section are defined as the ratio of this theoretical requirement to the energy actually supplied, referred to a target temperature of 720 °C throughout. The system boundary of the supplied energy is stated in each case, since figures with and without the energy required for cooling are not directly comparable.

(2) $$\Delta h_{\mathrm{Al},\mathrm{spec}}=\int_{T_{\mathrm{Ref}}}^{T_{m}}c_{p,\mathrm{Al},\mathrm{solid}}\left(T\right)\;dT+\Delta h_{\mathrm{fus}}+\int_{T_{m}}^{T_{\mathrm{target}}}c_{p,\mathrm{Al},\mathrm{liquid}}\left(T\right)\;dT$$

**Figure 5:**
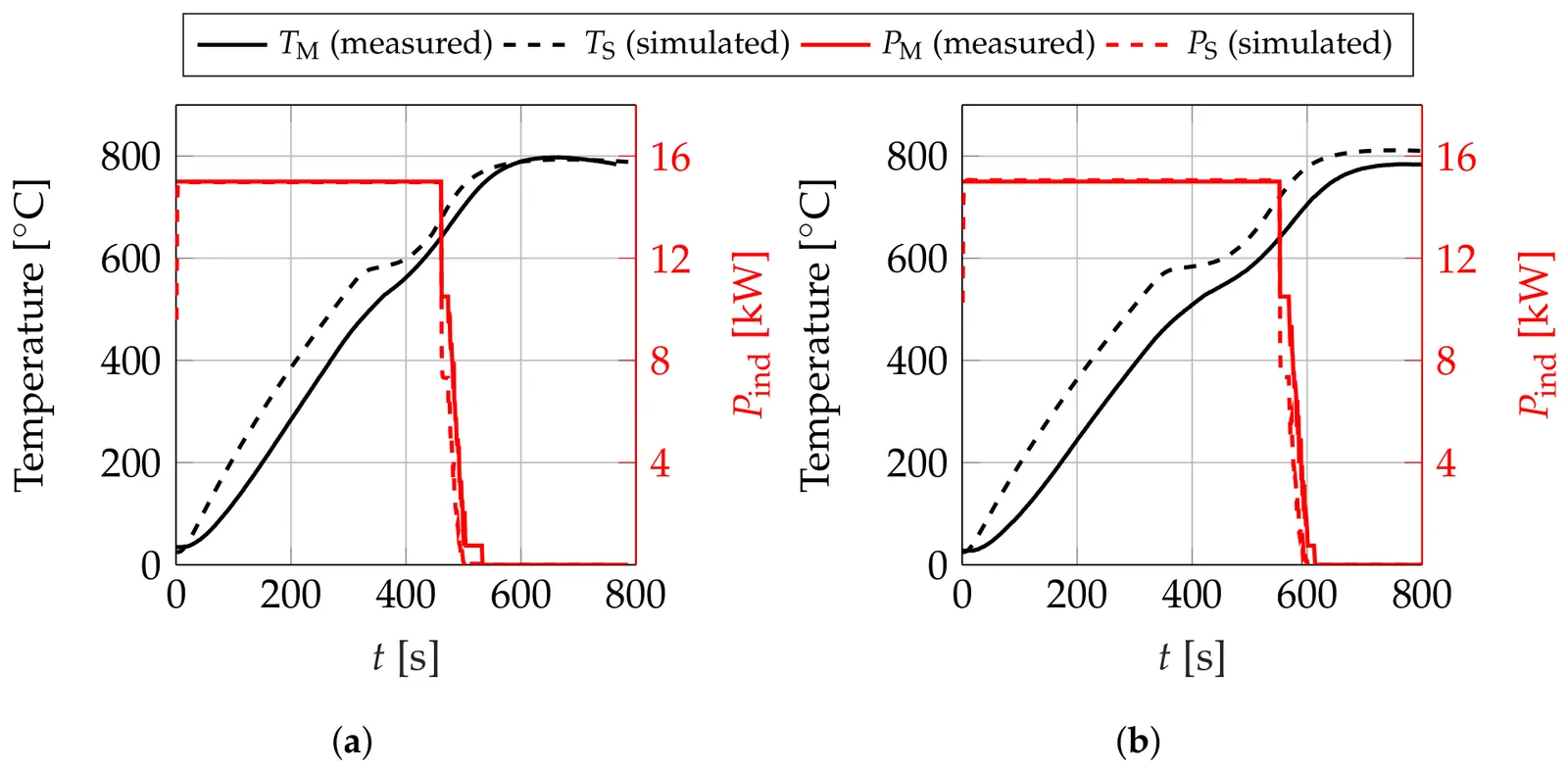
Measured and simulated crucible temperature and electrical power of the melting unit for different melt quantities. (**a**) 500 g. (**b**) 1120 g.

Here $\Delta h_{\mathrm{Al},\mathrm{spec}}$ is the specific theoretical enthalpy change during heating in J/kg, $T_{\mathrm{Ref}}$ the reference temperature at the start of heating with $T_{\mathrm{Ref}}=298.15$ K, $T_{m}$ the melting temperature and $T_{\mathrm{target}}$ the target temperature of the aluminum in K, $c_{p,\mathrm{Al},\mathrm{solid}}\left(T\right)$ and $c_{p,\mathrm{Al},\mathrm{liquid}}\left(T\right)$ the temperature-dependent specific heat capacities of solid and liquid aluminum in J/(kg K), and $\Delta h_{\mathrm{fus}}$ the latent heat of fusion in J/kg. The energy required for the experiment was determined from the power values recorded during the measurement series. The energy consumption of the melting unit was calculated using trapezoidal numerical integration of the power, with post-processing performed in MATLAB. Melting 500 g of aluminum alloy requires 1.96 kWh of energy, while melting 1120 g requires 2.36 kWh. The specific energy consumption is therefore 3920 kWh/t and 2107 kWh/t for the laboratory setup, giving efficiencies of 9.1% and 16.9% relative to the theoretical requirement. The energy for cooling is included in addition to the coil power. The power required for the cooler was taken from the data sheet and calculated from the melting time. The specific energy consumption for cooling is 562 kWh/t and 288 kWh/t for the laboratory setup. This gives a total specific energy consumption of 4482 kWh/t and 2395 kWh/t, and an overall efficiency of 7.9% and 14.9%. The gap between theoretical and measured energy requirements is due to several factors, including coil inefficiency, eddy current losses in surrounding components, heat losses to the environment, and the energy used by auxiliary systems such as cooling. In the laboratory setup, the crucible itself is also heated and, due to its larger volume, has a higher heat capacity than the alloy. This gap is expected to shrink with higher melting quantities. For larger plants and melting quantities, the numerical approach described in Section 2.2 is used for the energy assessment. First, the results are compared with the experimental data. Two experimental measurement series were simulated, as shown in Figure 5. The measured temperature ($T_{M}$), the simulated temperature ($T_{S}$), the measured power ($P_{M}$), and the simulated power ($P_{S}$) are compared. Minor deviations in the power are evident. For the temperature, the simulation rises earlier, while the measurement lags behind, most likely because the simplified model does not fully capture the thermal inertia of the overall system and of the temperature sensor. The total energy stored in the system at the end of the melting process nevertheless agrees with the simulation results. Because the transient behavior deviates, the model is regarded only as benchmarked against the measured power and temperature, and the scale-up results below are reported as predictions.

**Figure 6:**
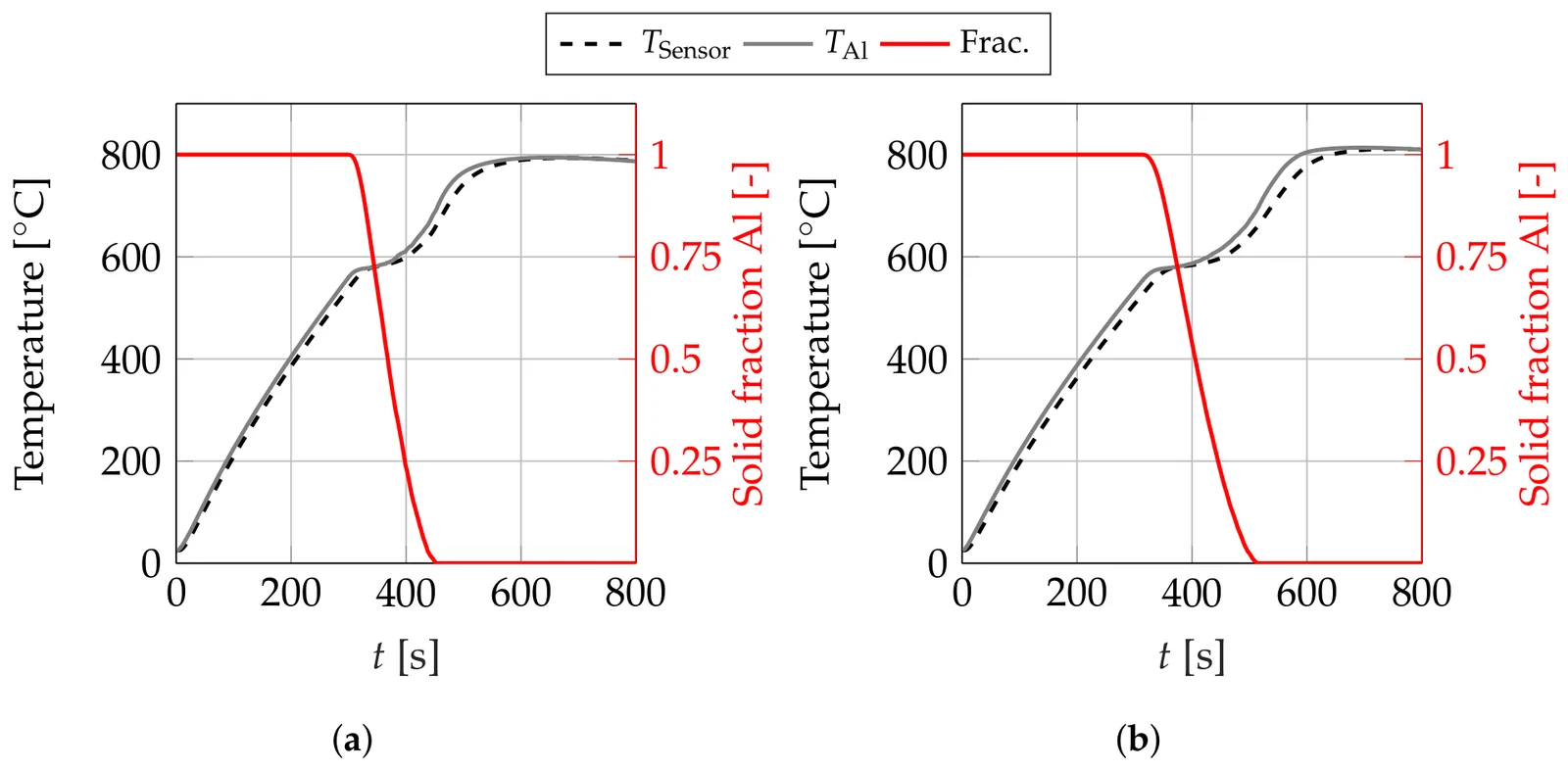
Comparison of the average aluminum temperature $T_{\mathrm{Al}}$ and the temperature at the sensor $T_{\mathrm{Sensor}}$ for different melt quantities, based on the simulation. The right ordinate gives the solid fraction of the aluminum (Frac.). (**a**) 500 g. (**b**) 1120 g.

The simulation also addresses a limitation of the experiment, namely that the thermocouple at the crucible base records the temperature at a single point only. Figure 6 compares the average simulated aluminum temperature with the sensor temperature over time for both measurements. The aluminum temperature runs slightly ahead of the sensor temperature and the two eventually converge, with deviations of up to 35 °C. The curves also show that the heating, melting, and superheating phases overlap in time, so the aluminum does not melt uniformly throughout the volume. Even where the temperature at the measuring point is above the melting temperature, the aluminum has not yet fully melted, as shown on the right ordinate, which gives the proportion of aluminum still solid. This has to be considered when single point measurements are interpreted.

**Figure 7:**
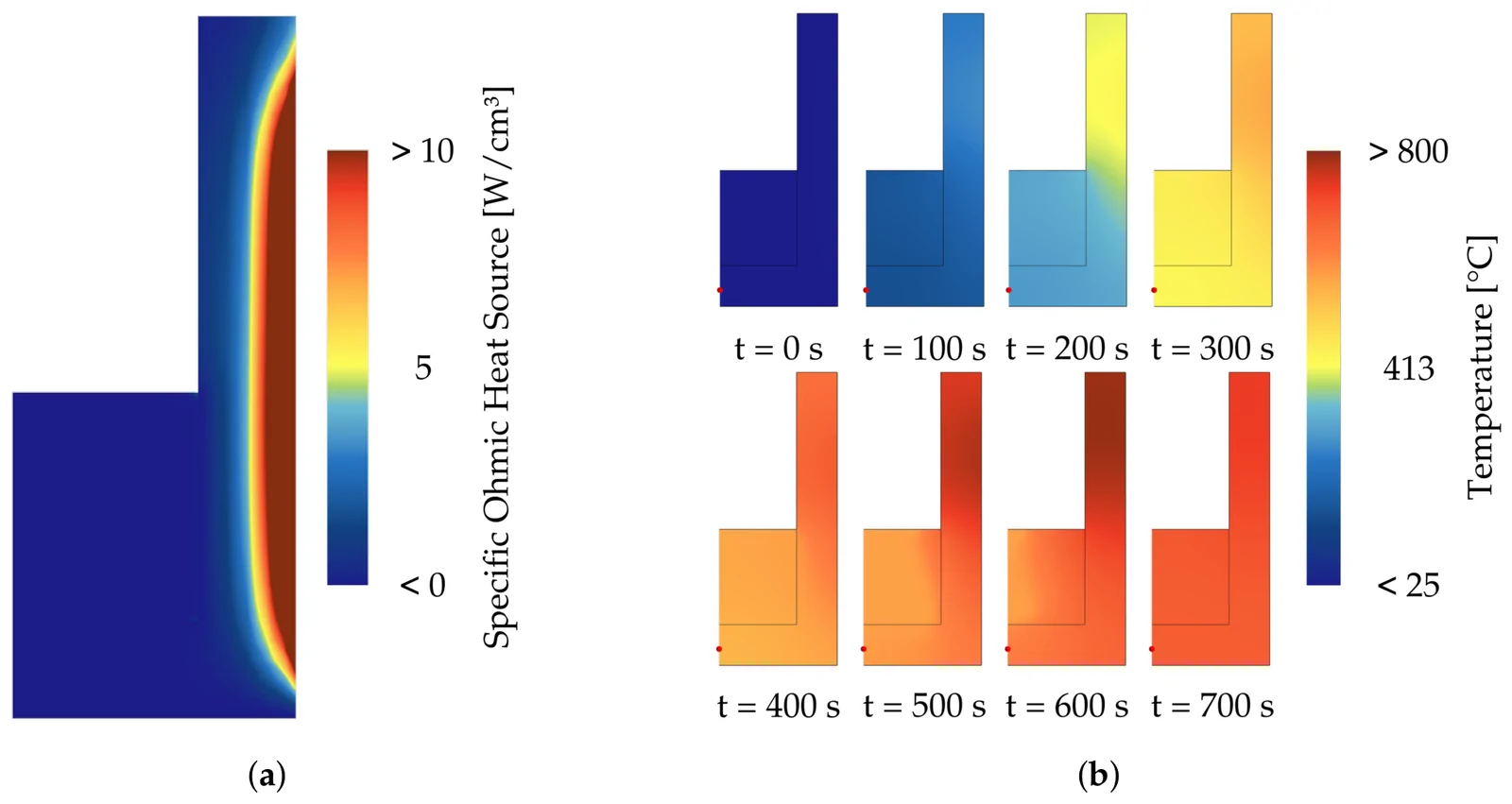
Energy input and temperature evolution on a center plane through the crucible and aluminum. (**a**) Specific ohmic heat source. (**b**) Transient temperature field for the 1120 g case.

The electrical power is not dissipated uniformly across the crucible and metal. Figure 7a shows the specific ohmic heat source in the crucible and in the aluminum. At the baseline operating frequency of 17.5 kHz, almost all the power is deposited in the graphite susceptor, with only 0.04% generated in the aluminum, consistent with the specification of the manufacturer that the coil primarily couples to the susceptor. As the frequency is lowered, the electromagnetic penetration depth increases and dissipation shifts into the metal: the aluminum share rises to 0.91% at 4 kHz, 5.79% at 1 kHz, and 76.35% at 50 Hz. At a sufficiently low frequency, most of the energy is therefore released directly into the metal, which also strengthens the electromagnetic body forces and can promote stirring of the melt. The concentration of power in the susceptor at the laboratory frequency also explains why the crucible absorbs a large share of the energy budget at small batch sizes, as quantified further below. A large industrial installation operating near line frequency would instead couple predominantly into the melt itself and avoid this penalty. The concentration of power in the susceptor governs how the temperature field develops. A mid-plane cross section through the crucible and the aluminum charge was extracted, and the transient temperature field visualized at 100 s intervals for the 1120 g case (*t* = 0–700 s), shown in Figure 7b.

This configuration is also analyzed in Figure 5b and Figure 6b. The temperature rise originates in the crucible and is then transferred to the aluminum by conduction and radiation. The rate and magnitude of this transfer are governed mainly by the effective interfacial area between crucible and metal available for heat exchange. Crucible regions without direct contact with the metal consistently show higher temperatures during heating, which points to local heat accumulation. Once the external heat input stops at *t* = 600 s, the remaining temperature gradients decay and the system relaxes toward a more homogeneous distribution as heat is redistributed by conduction within both components.

**Figure 8:**
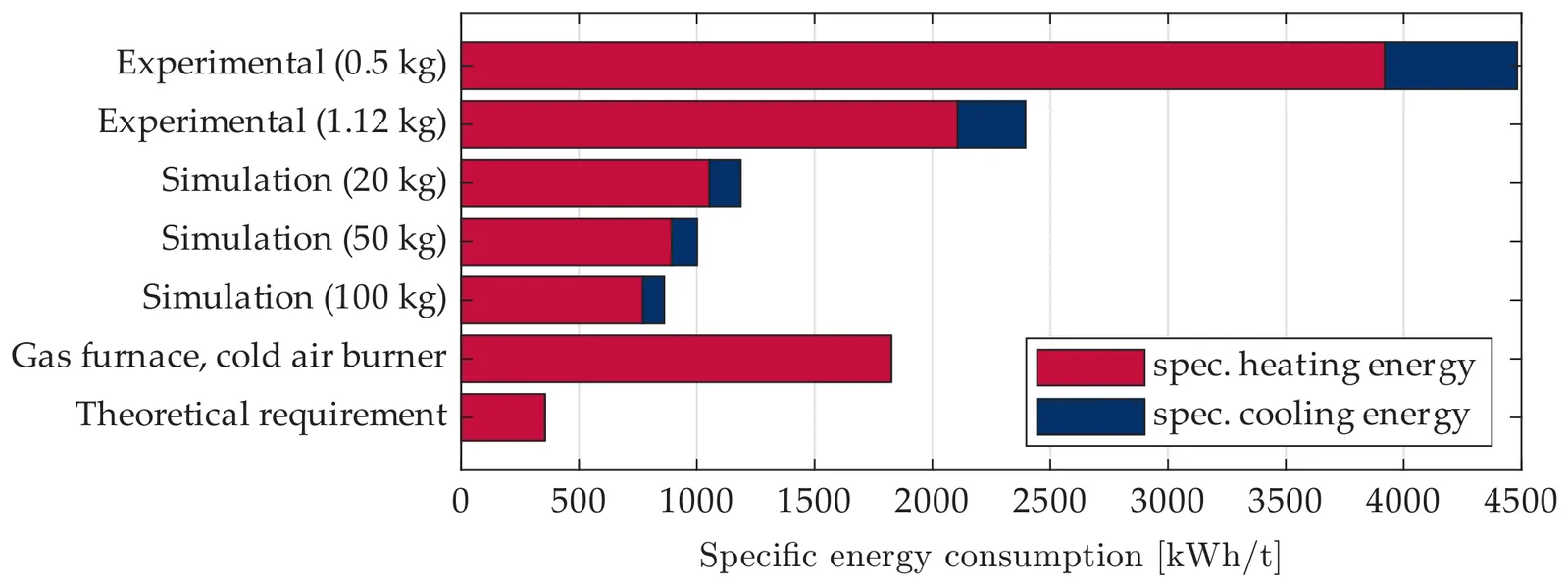
Comparison of the specific energy consumption for the measured and simulated scenarios and for reference values [6]. The theoretical requirement is the lower bound from Equation (2). The value for the gas furnace corresponds to the efficiency of 19.5% derived from [6], applied to the theoretical requirement from Equation (2).

Based on the benchmarked model, the process was scaled up to industrially relevant capacities. The electrical settings of the laboratory system, except for the power, were left unchanged. The heat loss at the system boundaries was converted to the surface area of the new scale, assuming comparable insulation and ambient conditions, and the cooling power was scaled to the new electrical power. Three scaling scenarios were considered, melting 20 kg, 50 kg, and 100 kg of aluminum with electrical powers of 30 kW, 50 kW, and 60 kW, respectively. The required energy was calculated by integrating the power input up to the point where the average aluminum temperature reached 720 °C. The specific energy consumption follows from dividing the total energy input by the respective aluminum mass (Equation (3)), and the theoretical energy requirement from Equation (2). A comparison of the predicted scenarios with reference values is shown in Figure 8.

(3) $$e_{\mathrm{spec}}=\frac{1}{m_{\mathrm{Al}}}\int_{0}^{t_{\mathrm{heat}}}P_{\mathrm{ind}}\left(t\right)\;dt$$

**Figure 9:**
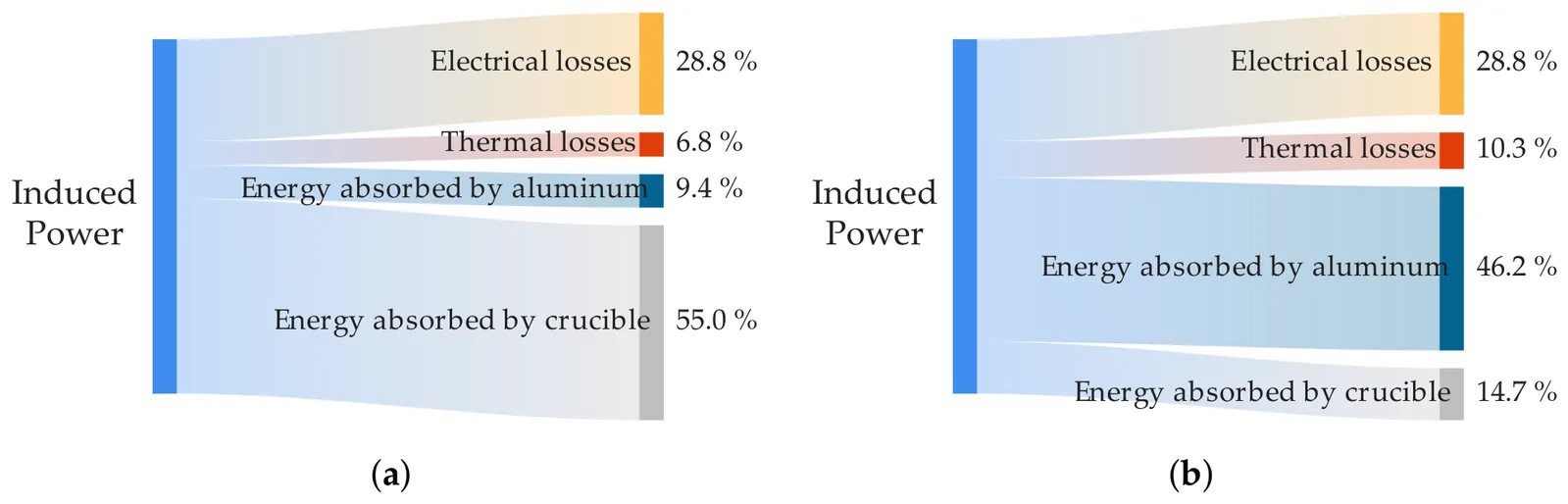
Energy distribution in the system for different melt quantities, based on the simulation. (**a**) 500 g. (**b**) 100 kg.

Here $e_{\mathrm{spec}}$ is the specific energy consumption for the heating process in J/kg, $m_{\mathrm{Al}}$ the mass of the aluminum to be heated in kg, $P_{\mathrm{ind}}\left(t\right)$ the time-dependent electrical power of the induction heating system in W, and $t_{\mathrm{heat}}$ the total heating time required to reach the target temperature, here 720 °C, in s. The energy analysis covers the melting phase only and does not include the subsequent holding phase. Within the melting phase, a further temperature rise is observed in the simulation after the target melt temperature is reached, which indicates additional thermal energy stored in the system. The scaled loss factors are transferred from the laboratory setup, whereas actual losses in industrial systems with optimized insulation may be lower. The efficiencies of 19.5% and 53.1% derived from [6] for gas-fired and induction furnaces (Section 1) are each related to the theoretical requirement given there, whereas the efficiencies in this work refer to the requirement from Equation (2). The measured laboratory values of 7.9% to 14.9% remain below the value for gas-fired furnaces, which reflects the crucible dominated energy budget at batch sizes of 500 g and 1120 g. Already for the smallest simulated batch size of 20 kg, however, the model predicts an efficiency of 33.8% without cooling and 30.0% with cooling, and therefore exceeds this value. Efficiency improves further with batch size, because the fixed thermal cost of heating the crucible is spread over more metal. Figure 9 compares the energy distribution when melting 500 g (Figure 9a) and 100 kg (Figure 9b). With small quantities, a large share of the energy goes into heating the crucible, and this share decreases as the melt quantity increases. For a melt quantity of 100 kg, the simulation predicts an efficiency of 46.2% relative to the induced power, and 41.3% including the energy for cooling. This compares with 53.1% derived for industrial induction furnaces. The difference is mainly due to the cold start of the simulation, which, as in the experiments, begins with the crucible at room temperature. According to Figure 9b, 14.7% of the induced power is used to heat the crucible. With a crucible already at operating temperature, the predicted efficiency rises to about 54% relative to the induced power and to about 48% including the energy for cooling. Since [6] does not state whether the energy for cooling and other auxiliary systems is included, the reference value lies within this range. The comparison is nevertheless only indicative, because the reference values are mean values from ten furnaces of unspecified size, recorded over longer periods of operation that include holding, charging and idle times [6], whereas the present balance covers the melting phase only. The assumptions listed in Section 2.2 can cause further small deviations.

In industrial operation, several factors lower the efficiency below that of the melting phase alone. The operating data in [6], which refer mainly to gas-fired reverberatory furnaces, show that the specific energy consumption rises sharply at low furnace utilization, since holding times for charging, casting, alloying and skimming add energy without increasing the amount of molten metal. Further factors identified in [6] are bath temperature setpoints above the required temperature, heat losses while the furnace is open for charging, and interruptions in manual charging. For a local unit, partial loading of the crucible, moist or oxidized charge material such as returns, and the wear of crucible and refractory act in the same direction. The electrical and thermal loss factors used here were transferred from the laboratory unit and may differ for an industrial installation. The operating frequency is a further design factor. The scaled models retain the laboratory frequency of 17.5 kHz, at which almost all of the power is dissipated in the graphite susceptor and reaches the metal only by heat transfer (Figure 7a). A unit operating at a lower frequency couples directly into the melt, so in this respect the predicted efficiencies are conservative.

### 3.2. Porosity Characterization in 2D

**Figure 10:**
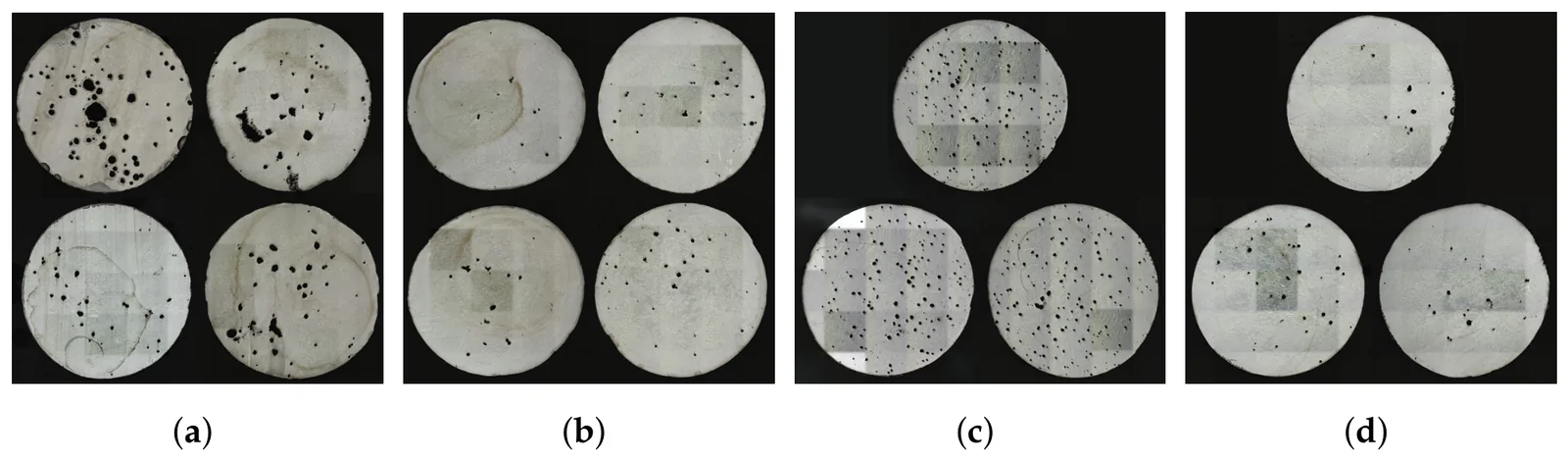
Sliced surfaces of specimens cast under different conditions. (**a**) Group T. (**b**) Group S. (**c**) R-01. (**d**) R-02.

The sliced surfaces of the casting trials were inspected and analyzed qualitatively to assess the impact of the processing conditions on porosity, using visual inspection and optical microscopy. The results are shown in Figure 10, where the subfigures are organized into the four groups T, S, R-01, and R-02 as defined in Table 1. Specimens from Group T show relatively larger pores. In the specimens of Group S, which also consist of virgin alloy but were treated with UST, porosity is lower and pores appear smaller and less frequent than in Group T, as shown in Figure 10a,b. The remelted specimens of R-01 display smaller but more numerous pores. When UST is applied to the remelted alloy in R-02, porosity is again lower and the pores become smaller, as shown in Figure 10d. The lower porosity of the specimens treated with UST is consistent with an effect of ultrasonic cavitation on the melt, which removes dissolved hydrogen and can also affect the oxide inclusions on which pores form, as discussed in Section 3.3. The hydrogen and inclusion contents were not measured in this study and the pores were not classified as gas or shrinkage porosity, so these attributions rest on the porosity data alone.

**Figure 11:**
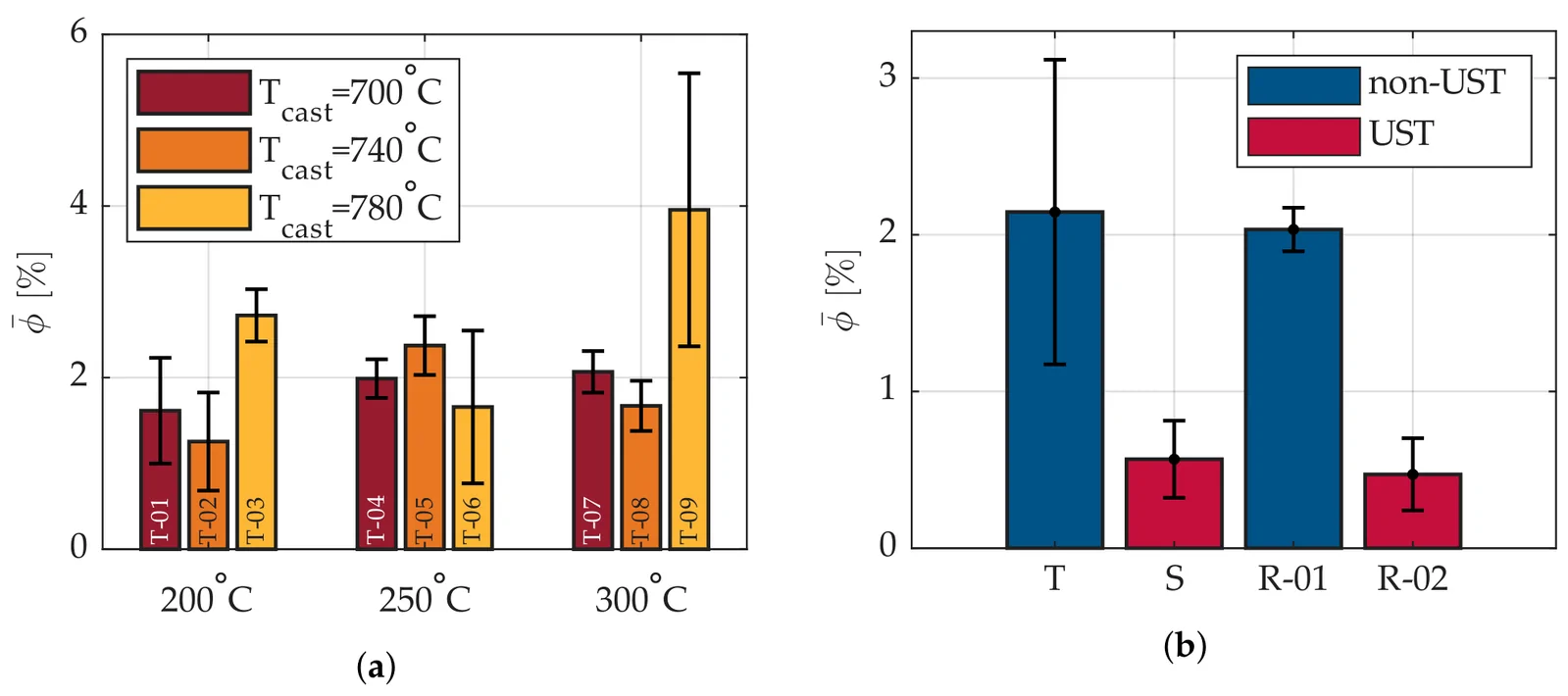
Mean porosity $\bar{\phi }$ of cast specimens under different conditions. (**a**) Specimens without UST, grouped by mold temperature $T_{\mathrm{mold}}$. (**b**) All types of specimens.

Porosity was quantified in MATLAB using the data files from ImageJ, as described in Section 2.1. The mean porosity $\bar{\phi }$ of the specimens cast without UST is shown in Figure 11a. For the first eight specimens $\bar{\phi }$ ranges from 1% to 3% and shows no clear trend with casting or mold temperature. Since each parameter combination was cast once, the absence of a trend does not show that porosity is insensitive to mold and casting temperature, as a small effect could be hidden by the variation between individual castings. Specimen T-09 is an exception, with a mean porosity of around 4% and a much larger standard deviation than the rest, possibly due to a single large pore. To compare specimens cast without UST with remelted specimens and with specimens cast with UST, the $\bar{\phi }$ values of Groups T and S are plotted alongside R-01 and R-02 in Figure 11b. For the specimens treated with UST, $\bar{\phi }$ is 0.56% against 2.14% without UST, with a lower standard deviation. The individual Group S specimens show no discernible difference in porosity across the sonication durations of 20 s, 40 s and 60 s. As each duration was cast once, this observation does not establish that the duration has no effect within this range, and the three castings were pooled into a single group for the further analysis on that basis. The remelted alloy without UST (R-01) showed a porosity of 2.03%, similar to the virgin untreated alloy but with a much smaller standard deviation. For the remelted alloy treated with UST (R-02), the mean porosity is 0.47%. Both the mean porosity and its standard deviation are therefore lower in the treated specimens, in virgin and in remelted alloys.

**Figure 12:**
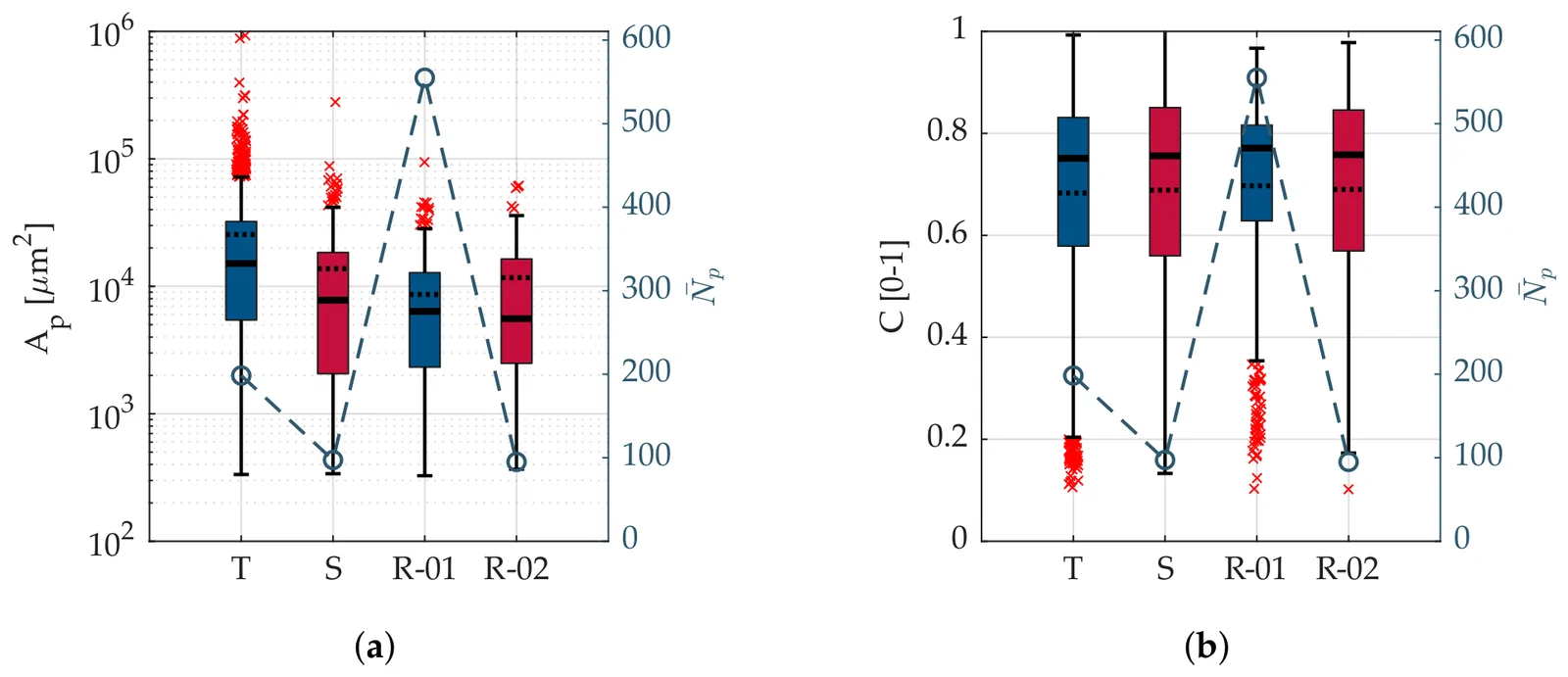
Pore size $A_{p}$ and circularity *C* for castings of groups T, S, R-01 and R-02. (**a**) Pore size. (**b**) Circularity. Red crosses (×) denote outliers beyond 1.5 times the interquartile range.

Figure 12a shows the pore size distributions $A_{p}$ for the AlSi12 castings across the four groups T, S, R-01, and R-02, using box plots on a logarithmic scale to accommodate the wide range of pore sizes. The box spans the 25th to the 75th percentile, the central line denotes the median and the dashed line the mean pore area, the whiskers extend to the most extreme values within 1.5 times the interquartile range, and values beyond are shown as outliers. The median pore area, a measure of typical pore size that is insensitive to outliers, is highest for Group T at 15,099 μm^2^, reflecting numerous large pores in untreated castings from virgin alloy. Group S, treated with UST, shows a reduced median of 7792 μm^2^. For remelted alloys, Group R-01 has a median of 6365 μm^2^, so that remelting alone yields smaller pores than in Group T. Group R-02, combining remelting and UST, has the lowest median at 5577 μm^2^. The secondary ordinate in Figure 12a shows the mean number of pores per specimen ${\bar{N}}_{p}$: T (198), S (97), R-01 (555), and R-02 (95). Groups S and R-02, both treated with UST, have far fewer pores than Group T. Possible causes are discussed together with the 3D results in Section 3.3. Group R-01 shows a markedly higher pore count despite a moderate median pore area, which indicates that remelting without UST is associated with a higher number of smaller pores. Since R-01 is a single casting, this increase has not been established as an effect of remelting. The increase is most likely caused by oxide films introduced with the remelted material. In crucible and induction melting, the surface oxide of the charge necessarily becomes part of the melt, and the strong electromagnetic stirring of an induction furnace keeps these oxides in suspension, so that they cannot separate by flotation or sedimentation [7]. Repeated remelting of 99.5% Al has accordingly been reported to degrade the ductility progressively, which is attributed to the increasing oxide content [7]. Oxide films act as initiation sites for pores, and the number of pores in a casting has been shown to depend on the number of oxide inclusions in the melt [19]. Additional hydrogen uptake during remelting may also contribute but cannot be assessed without measurements of the hydrogen content. The higher pore count at a similar overall porosity of 2.03% against 2.14% is, however, more consistent with additional initiation sites than with a higher hydrogen content, which would also be expected to enlarge the pores and increase the porosity. Within the pore population resolved by optical microscopy, the specimens treated with UST therefore show both a smaller median projected pore area and a lower pore count, while remelting alone is associated with a higher pore count. Section 3.3 shows that in the 3D evaluation the difference is carried almost entirely by the pore count, and discusses how the two evaluations relate. The comparable pore characteristics of S and R-02 indicate that remelted alloys treated with UST can reach a quality similar to virgin alloys. Figure 12b shows the circularity *C* of the pores, bounded between 0 and 1, where 1 is a perfect circle, across the four groups. Circularity is evaluated because rounded pores are typically associated with dissolved gas and irregular pores with shrinkage, although no clear separation between the groups emerged here. The median circularity of all groups is closely clustered at approximately 0.75, so that the typical pore shape differs little between groups. Group R-01 has the narrowest interquartile range, which indicates that most of its pores share a similarly high circularity, while Groups S and R-02 have the widest and thus show greater variability. Groups T and R-01 show numerous outliers below the lower whisker, which may reflect irregular pores formed by shrinkage or on entrained oxide films in the untreated alloys.

**Figure 13:**
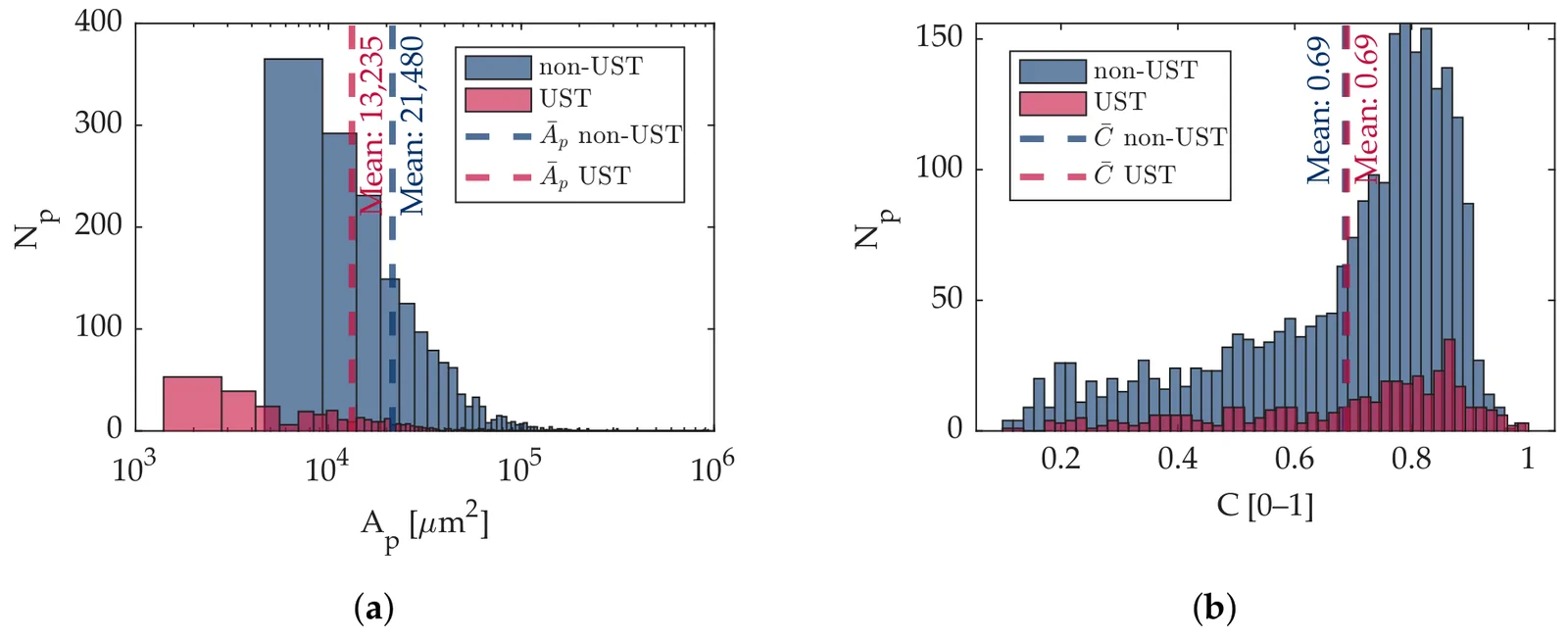
Pore size $A_{p}$ and circularity *C* for castings with and without UST. (**a**) Pore size. (**b**) Circularity.

Figure 13a shows histograms of pore size, overlaid with the mean value for each group. Most pores are small under both conditions, and the minimum pore size is lower in the castings treated with UST. The mean pore size for castings with UST is 13,235 μm^2^, compared with 21,480 μm^2^ without UST, a difference of 38%. Figure 13b shows histograms of pore circularity for the same groups. Both groups show the highest occurrence of pores around a circularity of 0.8, and the mean circularity is identical at 0.69, so that the average pore shape does not differ noticeably between the two conditions.

### 3.3. Porosity Characterization in 3D

**Figure 14:**
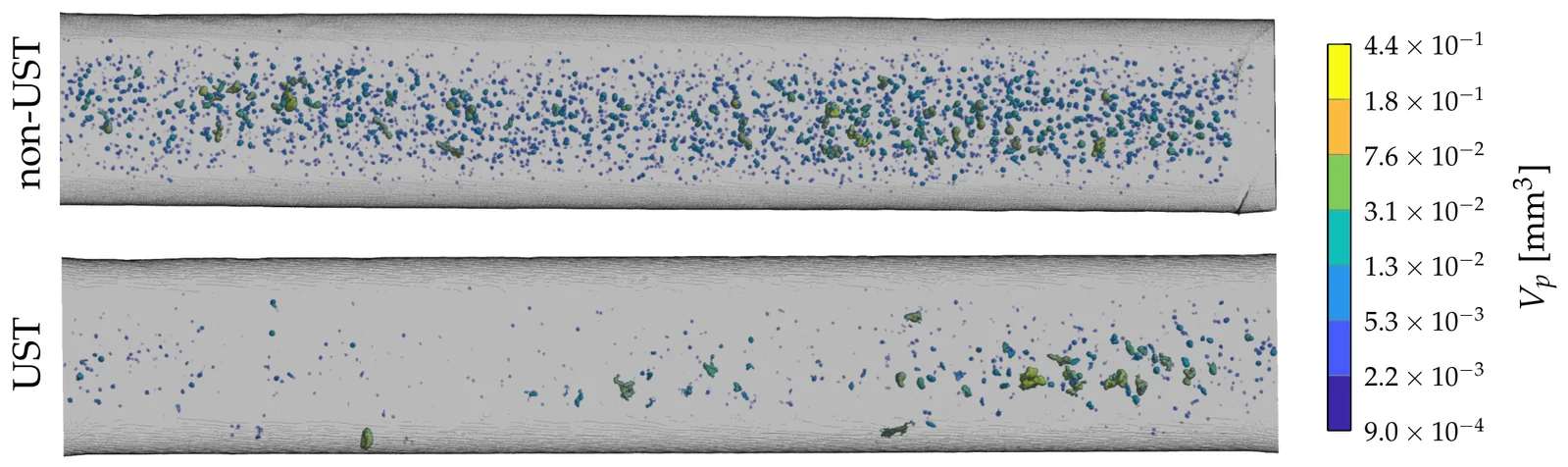
Reconstructed CT volumes of representative specimens without and with UST, with pores colored by volume $V_{p}$ on a shared logarithmic scale.

**Figure 15:**
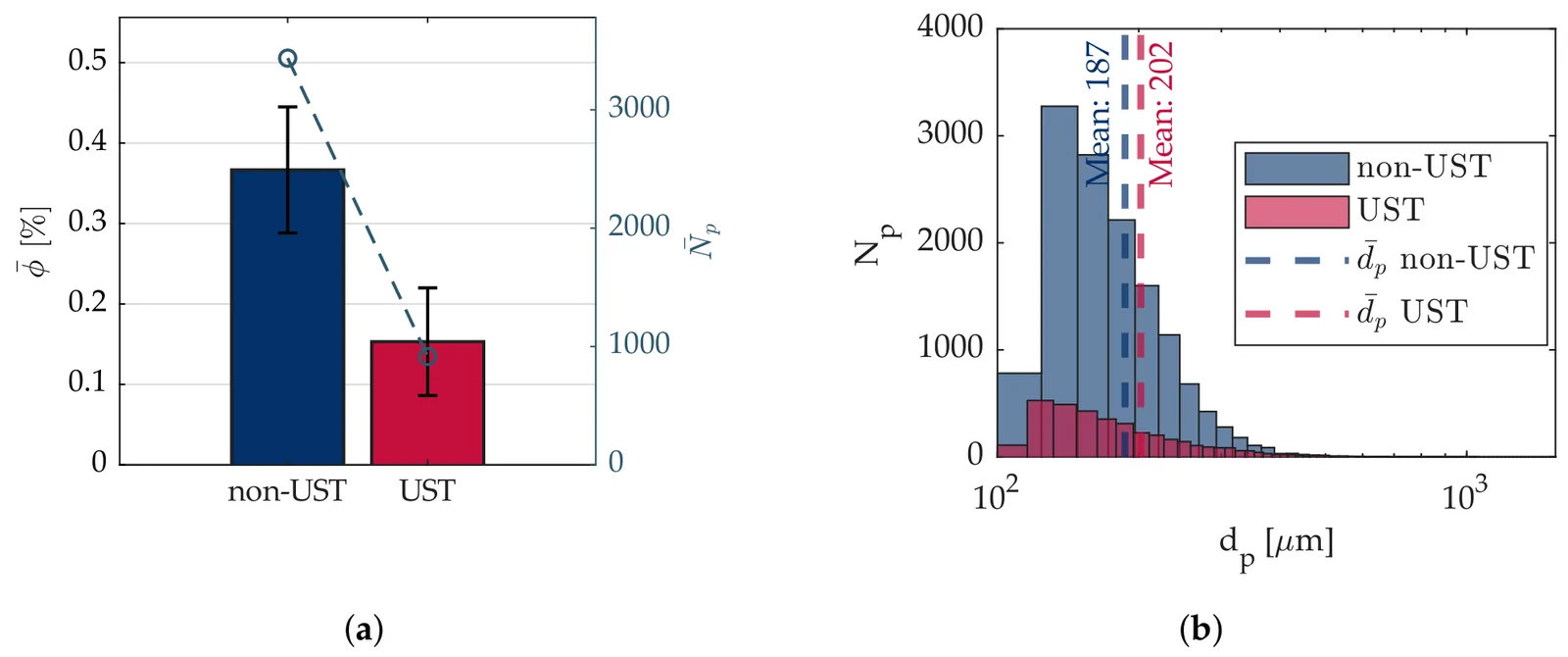
Volumetric porosity analysis of specimens without and with UST. (**a**) Mean porosity $\bar{\phi }$ and mean pore count ${\bar{N}}_{p}$. (**b**) Distribution of equivalent pore diameter $d_{p}$.

The analysis in Section 3.2 measures porosity on three polished cross sections per specimen and therefore samples only a small part of the specimen volume. To complement this, the eight CT scanned specimens of castings CT-01 to CT-04 as defined in Table 1 were evaluated volumetrically, which captures all pores above the resolution limit within the reference volume in 3D. Figure 14 shows reconstructed volumes for a specimen without and a specimen with UST. The specimen treated with UST contains fewer visible pores. This is reflected in the porosity values, shown in Figure 15a. Each value is given as mean ± standard deviation across n=4 specimens per group. The mean volumetric porosity $\bar{\phi }$ was 0.367±0.078% without UST and 0.153±0.067% with UST, a difference of 58.3%. Porosity ranged from 0.251% to 0.423% without UST and from 0.102% to 0.246% with UST, and the two ranges did not overlap. This is in line with the lower porosity found in 2D.

To examine whether this difference is associated with fewer pores, smaller pores, or both, pore count and pore volume were evaluated separately. The mean number of pores per specimen ${\bar{N}}_{p}$ was 3437 without UST and 915 with UST, a difference of 73.4%, shown on the right axis of Figure 15a. Pore size stayed very similar: the median pore volume $V_{p}$ was $2.66\times 10^{-3}\;{\mathrm{mm}}^{3}$ without UST and $2.82\times 10^{-3}\;{\mathrm{mm}}^{3}$ with UST. The distributions of equivalent pore diameter in Figure 15b also overlap closely, with medians of 171.9 μm without UST and 175.4 μm with UST. The mean diameters differ slightly more and are marginally higher for the treated specimens, at 187.5 μm and 202.4 μm. In 3D, the lower porosity of the specimens treated with UST results mainly from a smaller number of pores, while their size hardly changes. The two evaluations differ in this respect and should be read together. The analysis in 2D indicated smaller projected pore areas after UST, whereas the analysis in 3D showed largely unchanged equivalent pore diameters. Three differences between the evaluations contribute to this. First, the CT evaluation rejects all features below 20 voxels, corresponding to about 0.12 mm equivalent diameter, whereas the optical evaluation applies only a per image minimum area filter chosen to suppress noise, so that the two methods resolve different parts of the pore population. Second, a projected area on a random section and an equivalent diameter derived from a pore volume are different measures, and a section through a pore does not in general pass through its widest plane. Third, the specimens differ in material state, since the CT castings used 100% remelted alloy, whereas the values from 2D quoted for comparison come from castings with 50% remelted alloy. Grinding and polishing can additionally open subsurface pores and enlarge the apparent pore area on a section, which also contributes to the higher area fractions of 2.03% without and 0.47% with UST measured in 2D against the volume fractions reported above. Both methods consistently indicate a lower overall porosity and a lower pore occurrence for the specimens treated with UST. Several mechanisms can contribute to a lower pore count after UST. Pores in aluminum castings do not nucleate from dissolved hydrogen alone but form when hydrogen diffuses into entrained oxide films, so-called bifilms, and inflates them [19]. The number density of pores therefore follows the number of oxide inclusions in the melt, whereas the hydrogen content mainly determines how far these defects grow and thus the pore volume [19]. Within this concept, a lower pore count at almost unchanged pore size suggests that fewer oxide films were available for pore formation. UST can reduce the number of such films. Gas bubbles in the melt nucleate on oxide particles and carry part of them to the surface, and the collapse of cavitation bubbles has been proposed to fragment and disperse oxide films and to force the wetting of particle surfaces [26]. A wetted oxide fragment no longer offers the unbonded interface into which hydrogen can diffuse [19], so that such a treatment would reduce the number of potential pore sites without a change in the hydrogen content. Degassing can nevertheless contribute to the lower count, because with less dissolved hydrogen part of the oxide films may not grow to a resolvable size. As the CT evaluation rejects features below about 0.12 mm, close to the median equivalent diameter of about 0.17 mm, a general shift in the pore population towards smaller sizes would partly appear as a lower count of the resolved pores. The melt flow during treatment can act in both directions. The electromagnetic stirring of the induction unit keeps oxide inclusions in suspension [7], and acoustic streaming during UST adds to this flow, whereas the immersion and withdrawal of the sonotrode disturb the oxide skin on the melt surface and may entrain new films. Mold filling is not expected to cause a systematic difference, since castings with and without UST were poured into the same mold in the same way, but it adds to the variation between castings (Section 3.5). Without measurements of the hydrogen and inclusion contents, these effects cannot be separated, and the lower pore count is attributed to the combined action of UST on the melt.

The literature values cited in Section 1 can only partly be compared with the present results. The reduction in the hydrogen content from 0.45 ppm to 0.14 ppm within one minute was obtained in 0.2 kg of A356 at 740 °C [21]. In the same study, the time needed to reach the steady-state hydrogen content at 700 °C increased with the melt mass from about 1 min for 0.2 kg to about 4 min for 0.6 kg [21]. With 500 g and sonication times of 20 s to 60 s, the present treatment was therefore probably shorter than required to reach this state, and since the hydrogen content was not measured, a reduction comparable to [21] cannot be assumed. The density of 98.5% of the theoretical value in [23] and the hydrogen contents in [21], which were derived from density measurements, refer to samples solidified under reduced pressure, which enlarges the pores in order to make the gas content visible, whereas the porosities reported here were measured on castings solidified at ambient pressure. The mean porosities of 0.56% in 2D and 0.153% in CT after UST correspond to relative densities above 99%, but because of the different methods these values do not indicate a more effective treatment than in [23]. A closer comparison is possible with a study that used the same type of ultrasonic unit and also evaluated the porosity by CT, in which UST of A356 directly before tilt casting reduced the total pore volume by 53%, with almost all remaining pores smaller than 0.02 mm^3^ [24]. The reduction in the volumetric porosity by 58.3% found here is of similar magnitude, but in the present work it resulted from a lower pore count at almost the same pore size. This agrees with the interpretation in the preceding paragraph.

### 3.4. Tensile Properties

**Figure 16:**
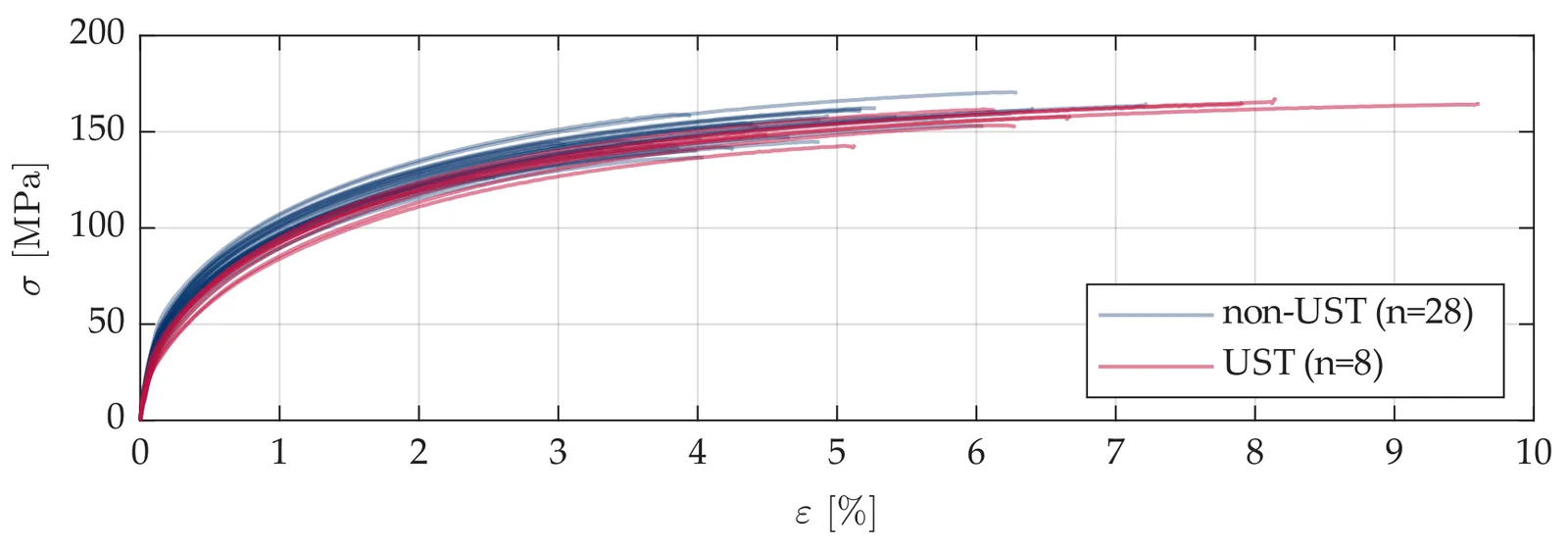
Stress-strain curves of all tensile tested specimens, grouped by UST condition.

**Figure 17:**
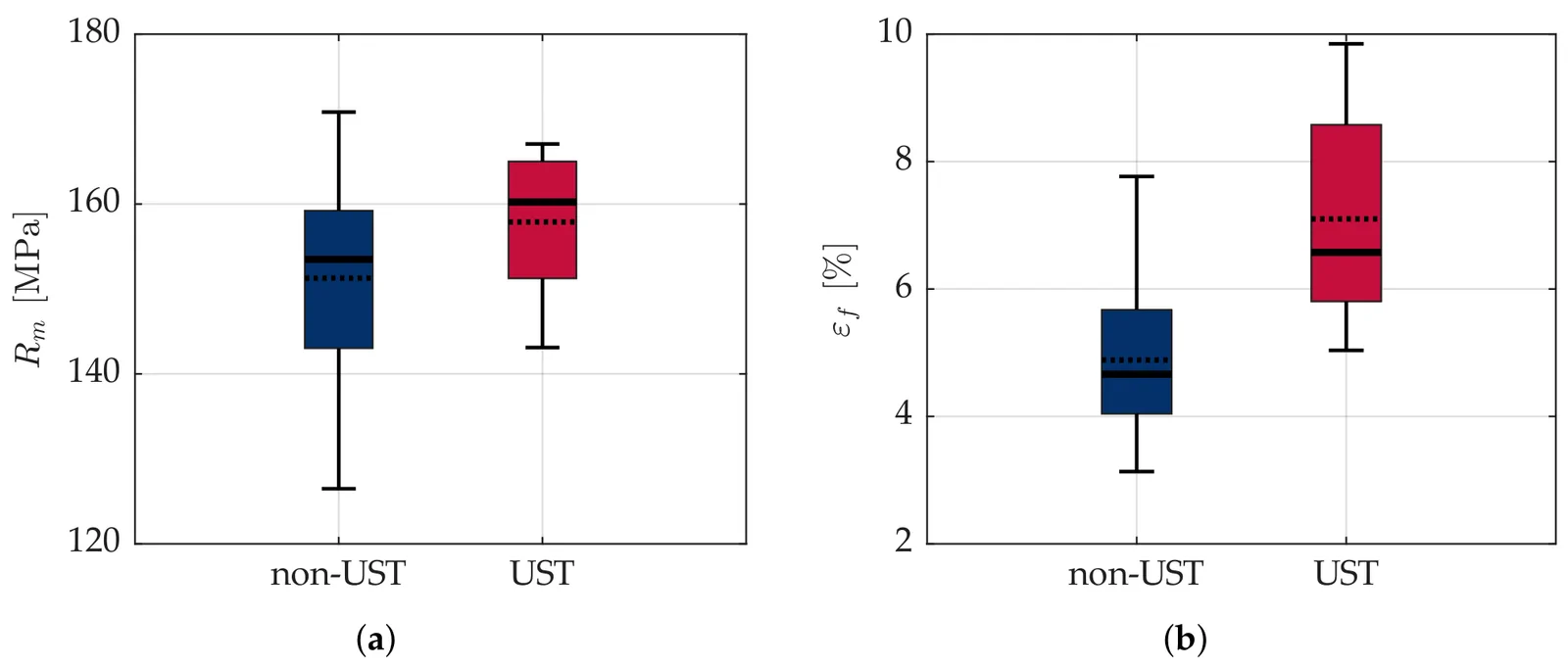
Tensile properties of specimens. (**a**) Ultimate tensile strength $R_{m}$. (**b**) Strain at fracture $\varepsilon_{f}$.

Tests in which fracture occurred outside the gauge length were excluded from the evaluation, leaving 36 valid tests. This excluded 2 of 30 tests without UST and 4 of 12 tests with UST. Specimens are grouped by UST condition, pooling virgin and 50% remelted alloy: the group without UST comprises Groups T and R-01 (n=28), the group with UST comprises Groups S and R-02 (n=8). The two groups therefore differ both in size and in the process conditions they contain, and the comparison below is descriptive. Figure 16 shows the stress-strain curves of all tested specimens, and Figure 17 summarizes the ultimate tensile strength $R_{m}$ and the strain at fracture $\varepsilon_{f}$ for both conditions.

The ultimate tensile strength was 151.3±10.3 MPa without UST and 157.9±8.6 MPa with UST, so that the specimens treated with UST exhibited a 4.4% higher mean strength. Their mean strain at fracture was 45.5% higher, at 7.10±1.72% against 4.88±1.14%, and their mean toughness, taken as the area under the stress-strain curve, was 53.2% higher, at 9.13±2.89 MJ/m^3^ against 5.96±1.82 MJ/m^3^. The distributions of the two conditions overlap for all three metrics and are shifted against each other. The differences were tested with Welch’s *t*-test, which does not assume equal variances or equal group sizes. The difference in mean tensile strength of 6.6 MPa is smaller than the standard deviation within each group and is not statistically significant (p=0.09). The differences in strain at fracture (p=0.008) and toughness (p=0.02) are significant at the 5% level. These *p* values treat the individual specimens as independent observations. Since the specimens from one casting are not independent and the group treated with UST comprises only four castings, the *p* values overstate the statistical confidence and are used only to rank the differences. The increase in tensile strength is much smaller than the 50% reported for AlSi9Cu3, where UST raised the ultimate tensile strength from 220 MPa to 332 MPa and the strain from 0.6% to 2.9% [22]. This improvement was attributed to the refinement of the α-Al grains and of the intermetallic phases, obtained by sonicating 0.4 kg of melt isothermally between 3 K and 28 K above the liquidus temperature immediately before pouring [22]. Refinement by UST is usually carried out slightly above the liquidus, so that the nuclei created by cavitation survive until solidification, and the effect fades when the melt is reheated [23], whereas degassing proceeds fastest at around 700 °C [21]. The present treatment at 700 °C, about 120 K above the liquidus of AlSi12, followed by 2 to 3 min before pouring, corresponds to these degassing conditions, so that a refinement of the extent reported in [22] is not expected. In addition, the near-eutectic AlSi12 contains only a small fraction of primary α-Al that could be refined. The present results are closer to studies that treated the melt well above the liquidus. A study with the same type of ultrasonic unit on A356 reported an increase in tensile strength from 170.1 MPa to 179.1 MPa (5%) and in elongation from 4.7% to 5.5% (17%) [24], and contactless UST of commercial purity aluminum with a small grain refiner addition at 710 °C raised the tensile strength by about 17% and the elongation by 52% to 73%, the latter only in combination with grain refinement [26]. The present increase in strength of 4.4% is comparable to [24], and the increase of 45.5% in strain at fracture lies between the values of [24,26].

The difference between the two groups is larger for ductility than for strength, and the strain at fracture also varies considerably more between specimens than the tensile strength, as shown in Figure 17. Both observations are typical of fracture controlled by casting defects. In a cast Al–7Si–Mg alloy, fracture has been shown to initiate mostly at shrinkage pores and oxide films [29], and a similar role of pores can be expected in AlSi12. With fewer pores, cracks would initiate later, so the material could deform plastically to a larger extent before failure. A refinement of the microstructure is a further possible cause. In [26], a treatment that only degassed the melt left the elongation unchanged, so that its increase was attributed to the grain refinement. Since neither the fracture surfaces nor the microstructure were examined here, the contributions of fewer pores and of a possible refinement to the higher ductility cannot be separated.

### 3.5. Limitations

Given the pilot scale design of this study, the results should be interpreted descriptively. Each parameter combination in Groups T, S and R was cast once, so a small effect of mold temperature, casting temperature or sonication duration could be hidden by the variation between individual castings. The analysis of porosity in 2D is based on three sections from one specimen per casting and uses image specific segmentation thresholds, which introduces a dependence on operator judgment. The tensile comparison pools several process conditions into two groups and contains unequal numbers of valid specimens, and the specimens within one casting are not independent replicates. The comparison by computed tomography comprises four specimens per condition from two castings, and these specimens were cast from 100% remelted alloy, whereas the values from 2D used for comparison came from castings with 50% remelted alloy. The two methods also resolve different parts of the pore population, as set out in Section 3.3. Neither the hydrogen content nor the inclusion content of the melt was measured, and the pores were not classified as gas or shrinkage porosity, so degassing and an effect on oxide inclusions are interpretations of the porosity data that have not been demonstrated directly. The microstructure was not examined. Since UST above the liquidus has been shown to refine the grain size of commercial purity aluminum by up to 48% [26], a refinement that improves feeding and reduces shrinkage porosity cannot be excluded as a further contribution. Only one alloy was investigated. The melt was poured by hand, and although the pouring time was approximately 3 to 4 s per casting, the pouring rate was not controlled, so mold filling and the amount of entrapped air and oxide films may have differed between castings, which increases the variation in the results. Since castings with and without UST were poured in the same way, no systematic bias between the two conditions is expected, but with one casting per parameter combination, random differences in pouring can still contribute to the differences between individual castings. The efficiencies reported for industrial scale are model based estimates for the melting phase only, exclude holding, and rest on loss factors transferred from the laboratory setup rather than measured on an industrial installation. The results therefore support the feasibility of the combined approach, but they do not establish statistically independent causal effects of individual process parameters or full scale process performance.

### 3.6. Industrial Deployment

This work considers local melting units mounted directly on casting machines, which requires melting smaller quantities of alloy. Section 3.1 indicates that a workable efficiency can be reached even at a small melt quantity of around 20 kg, and that this efficiency improves further as batch size increases toward the range of 50 kg to 100 kg relevant to a casting cell. Local decentralized units also allow flexible operation, since alloys can be produced on demand and thermal losses from transporting liquid aluminum between a central furnace and each casting station can be reduced. Induction melting is inherently a batch process, so a configuration with two units per casting cell may be used to keep a casting line continuously supplied: one unit melts a new batch while the other holds the previous batch ready for casting, and the two operate in alternation [30]. Such an arrangement can avoid interruptions and keep throughput stable. A single compact unit can combine melting, melt treatment, and holding, since UST requires no consumables, gas, or rotating hardware and can be applied directly in the same crucible used for melting. In a decentralized arrangement, rotary degassing would require a gas supply and a rotor at every casting station, whereas the ultrasonic unit used here fits within the space of the melting unit and introduces no process gas. The porosity and tensile results in Section 3.2, Section 3.3 and Section 3.4 suggest that an integrated treatment step of this kind may reduce the need for a separate degassing station, subject to the limitations set out in Section 3.5.

## 4. Conclusions

This pilot study examined local induction melting combined with UST as a decentralized and electrified alternative to centralized gas-fired melting for AlSi12 castings. Within the limits of a pilot study with one alloy and one casting per parameter combination, it shows that the combined process is feasible and gives first indications of its effects. Including the energy for cooling, the measured efficiency at laboratory scale was 7.9% to 14.9%, as most of the energy heats the crucible at small batch sizes. The benchmarked simulation predicts 30.0% for a batch of 20 kg, already above the 19.5% derived for gas-fired furnaces, and 41.3% for 100 kg, both including the energy for cooling. With a crucible already at operating temperature, the prediction for 100 kg rises to about 48% with and 54% without the energy for cooling, which brackets the 53.1% derived from operating data of industrial induction furnaces. These figures cover the melting phase only, so holding and idle times will lower the efficiency in operation. UST lowered the mean porosity in both evaluations, from 2.14% to 0.56% in 2D and from 0.367% to 0.153% in CT. In CT, this was associated with a 73% lower pore count at an essentially unchanged pore size, which is consistent with a lower number of oxide inclusions on which pores form as well as with degassing, two effects that cannot be separated without measurements of the melt. In the single casting from 50% remelted alloy without UST, the pore count was almost three times that of virgin alloy, most likely because of oxide films introduced with the remelted material, whereas remelted alloy treated with UST reached a pore count similar to that of treated virgin alloy. The specimens treated with UST showed a 45.5% higher mean strain at fracture and a 53.2% higher mean toughness, whereas the 4.4% higher mean tensile strength was not statistically significant. UST therefore mainly affects the ductility, although the pooled comparison with only four treated castings limits the certainty of this conclusion. Because UST requires neither consumables nor a gas supply, it can be integrated into a compact decentralized melting unit alongside induction melting. Further work should measure the hydrogen and inclusion contents of the melt, so that degassing can be separated from the effect of UST on oxide inclusions, examine the fracture surfaces to identify the defects at which fracture initiates, and replicate each parameter combination often enough to resolve the influence of mold temperature, casting temperature and sonication duration. Transferring the energy analysis to an industrial installation would replace the loss factors scaled from the laboratory setup with measured values and allow the holding phase to be included.

## Figures and Tables

**Table 1:** Table of experiments. Each row corresponds to a single casting, except for the two CT rows, which each comprise two replicate castings.

ID	$T_{\mathrm{mold}}$ [°C]	$T_{\mathrm{cast}}$ [°C]	$t_{\mathrm{UST}}$ [s]	*R* [%] ^a^
T-01	200	700	0	0
T-02	200	740	0	0
T-03	200	780	0	0
T-04	250	700	0	0
T-05	250	740	0	0
T-06	250	780	0	0
T-07	300	700	0	0
T-08	300	740	0	0
T-09	300	780	0	0
S-01	200	700	20	0
S-02	200	700	40	0
S-03	200	700	60	0
R-01	200	700	0	50
R-02	200	700	60	50
CT-01, CT-02	200	700	0	100
CT-03, CT-04	200	700	20	100

^a^ Percentage of remelted AlSi12 alloy used in the casting.

**Table 2:** Thermophysical properties of AlSi12 and graphite as a function of temperature. Graphite data from [28], AlSi12 data from Thermo-Calc 2024b (Al Databank 4.0).

Property	Material	Temperature [°C]					
		25	200	400	600	800	1000
heat capacity $c_{p}$ [J/(kg K)]	AlSi12	884	959	1048	1117	1149	1150
	Graphite	734	1155	1486	1697	1835	1929
thermal conductivity λ [W/(m K)]	AlSi12	203	190	187	60	72	77
	Graphite	150	163	170	167	159	150

## Data Availability

The data presented in this study are available on request from the corresponding authors (the data are not publicly available due to restrictions of the funding project).
